# Prostate cancer cell-platelet bidirectional signaling promotes calcium mobilization, invasion and apoptotic resistance via distinct receptor-ligand pairs

**DOI:** 10.1038/s41598-023-29450-x

**Published:** 2023-02-17

**Authors:** Kaitlin Garofano, Kameron Rashid, Michael Smith, Christine Brantner, Sumanun Suwunnakorn, David Diemert, Olivia Gordon, Anelia Horvath, Sikandar Khan, Anastas Popratiloff, Johng Rhim, Alfateh Sidahmed, Sanjay B. Maggirwar, Travis J. O’Brien, Minoli A. Perera, Norman H. Lee

**Affiliations:** 1 Department of Pharmacology and Physiology, George Washington University, 2300 I Street NW, Washington, DC 20037 USA; 2 GW Nanofabrication and Imaging Center, The George Washington University, Washington, DC 20037 USA; 3 Department of Microbiology Immunology and Tropical Medicine, The George Washington University, Washington, DC 20037 USA; 4 Department of Medicine, George Washington University, Washington, DC 20037 USA; 5 Department of Biochemistry and Molecular Medicine, George Washington University, Washington, DC 20037 USA; 6 Department of Surgery, Uniformed Services University of the Health Sciences, Bethesda, MD 20814 USA; 7 Department of Pharmacology and Center for Pharmacogenomics, Northwestern University, Chicago, IL 60611 USA; 8 GW Cancer Center, George Washington University, Washington, DC 20037 USA

**Keywords:** Cell biology, Computational biology and bioinformatics, Molecular biology, Cancer, Cancer genomics, Cancer microenvironment, Tumour biomarkers, Urological cancer

## Abstract

Platelets play a crucial role in cancer and thrombosis. However, the receptor-ligand repertoire mediating prostate cancer (PCa) cell-platelet interactions and ensuing consequences have not been fully elucidated. Microvilli emanating from the plasma membrane of PCa cell lines (RC77 T/E, MDA PCa 2b) directly contacted individual platelets and platelet aggregates. PCa cell-platelet interactions were associated with calcium mobilization in platelets, and translocation of P-selectin and integrin α_IIb_β_3_ onto the platelet surface. PCa cell-platelet interactions reciprocally promoted PCa cell invasion and apoptotic resistance, and these events were insensitive to androgen receptor blockade by bicalutamide. PCa cells were exceedingly sensitive to activation by platelets in vitro, occurring at a PCa cell:platelet coculture ratio as low as 1:10 (whereas PCa patient blood contains 1:2,000,000 per ml). Conditioned medium from cocultures stimulated PCa cell invasion but not apoptotic resistance nor platelet aggregation. Candidate transmembrane signaling proteins responsible for PCa cell-platelet oncogenic events were identified by RNA-Seq and broadly divided into 4 major categories: (1) integrin-ligand, (2) EPH receptor-ephrin, (3) immune checkpoint receptor-ligand, and (4) miscellaneous receptor-ligand interactions. Based on antibody neutralization and small molecule inhibitor assays, PCa cell-stimulated calcium mobilization in platelets was found to be mediated by a fibronectin1 (FN1)-α_IIb_β_3_ signaling axis. Platelet-stimulated PCa cell invasion was facilitated by a CD55-adhesion G protein coupled receptor E5 (ADGRE5) axis, with contribution from platelet cytokines CCL3L1 and IL32. Platelet-stimulated PCa cell apoptotic resistance relied on ephrin-EPH receptor and lysophosphatidic acid (LPA)-LPA receptor (LPAR) signaling. Of participating signaling partners, FN1 and LPAR3 overexpression was observed in PCa specimens compared to normal prostate, while high expression of CCR1 (CCL3L1 receptor), EPHA1 and LPAR5 in PCa was associated with poor patient survival. These findings emphasize that non-overlapping receptor-ligand pairs participate in oncogenesis and thrombosis, highlighting the complexity of any contemplated clinical intervention strategy.

## Introduction

While platelets are primarily known for their cardiovascular role in thrombosis and hemostasis, increasing evidence points to their critical role in cancer progression and mortality. Platelets have been proposed to form a physical barrier around circulating tumor cells (CTCs) in the bloodstream, leading to tumor cell evasion of the immune system and protection against shear stress^1–3^. Interactions between CTCs and platelets also give rise to bidirectional signaling through direct transmembrane ligand and receptor coupling and/or indirect paracrine signaling events^4^. Platelet-stimulation of CTCs appears to be critical for the metastatic programming of CTCs such as the ability to intravasate, extravasate and colonize to distal sites^4,5^. Conversely, tumor cell-stimulated platelet aggregation (TCIPA) has been proposed to contribute to thrombosis and venous thromboembolism (VTE). It is estimated that up to 20% of cancer patients suffer from VTE^6^, and 60–75% of occult cancers are detected within a year of diagnosing idiopathic VTE^7^. High platelet counts are associated with increased cancer metastasis and worse prognosis^8–11^. Interfering with bidirectional signaling events during platelet-cancer cell interactions has been shown to decrease platelet activation and suppress cancer metastasis. For example, preventing platelet surface protein P-selectin from ligating with P-selectin glycoprotein ligand-1 (PSGL1) on lung cancer cells^12^ or mucins on colon cancer cells^1^ reverses TCIPA and diminishes metastasis, respectively. Conversely, abrogating binding of podoplanin found on the surface of melanoma, breast cancer, lung cancer and osteosarcoma cells to C-type lectin-like receptor 2 (CLEC-2) on platelets has been shown to reduce TCIPA in vitro and VTE in mouse models^13–16^.

Prostate cancer (PCa) is the most common cancer and the second most common cause of cancer-related deaths among men in the United States^17^. Worldwide, PCa is the fifth leading cause of cancer-related deaths in men^18^. Despite in-depth studies elucidating the cell autonomous mechanisms of PCa progression and metastasis^19^, an understanding of the functional consequences of PCa cell-platelet interactions is fragmentary and many questions remain. For example, platelet-derived microparticles containing cytokines and growth factors are known to increase invasion of the PCa cell line Cl-1^20,21^. The identity of these cytokines and growth factors has yet to be fully ascertained, but appears not to be vascular endothelial growth factor, platelet-derived growth factor (PDGF) or basic fibroblast growth factor^20,21^. Platelet interactions with the PCa cell line PC-3 ectopically over-expressing mutant Ras have been shown to facilitate both TCIPA and metastatic programing through an ADGRE5 (on cancer cell surface)- and integrin α_IIb_β_3_ (on platelet surface)-dependent process, albeit these two surface signaling proteins do not appear to be direct binding partners^22^. Collectively, these preliminary studies pose intriguing questions on the nature of the direct binding partners mediating PCa cell-platelet crosstalk, and whether platelets have the capacity to promote additional hallmarks of cancer in PCa cells besides metastatic programming (e.g. proliferation, apoptotic resistance, avoiding immune destruction).

In this study, we used immunofluorescence and transmission electron microscopy to quantify and characterize the ultrastructural interactions between platelets and two PCa cell lines, MDA PCa 2b and RC77 T/E. We demonstrate that platelets in a concentration-dependent manner promote not only tumor cell invasion but also apoptotic resistance. Moreover, we establish that calcium mobilization, invasion and apoptotic resistance resulting from PCa cell-platelet interactions are each mediated by distinct receptor-ligand pairings. Particularly noteworthy, many of the functionally validated signaling partners on the PCa side (comprising various classes of receptors and ligands) were either over-expressed in PCa compared to normal prostate or associated with worse PCa patient survival based on publicly available RNA-Seq data in The Cancer Genome Atlas (TCGA). Taken together, our findings underscore the relevance of PCa cell-platelet interactions.

## Materials and methods

### Washed platelet isolation from whole blood samples

Whole blood was collected from healthy individuals in compliance with an approved George Washington University (GWU) Internal Review Board protocol (IRB approval number 021747). All methods and experimental protocols were carried out in accordance with IRB guidelines and regulations. Informed consent was obtained from all subjects. Platelet-rich plasma (PRP) was isolated by spinning whole blood at 170× *g* for 15 min. Two hundred and fifty nM prostaglandin E1 and 250 mM EDTA were added to the PRP prior to centrifugation at 800× *g* for 15 min. After removal of the supernatant, the resulting platelet pellet was washed in 4 ml of phosphate buffered saline (PBS) containing 250 nM PGE1. Platelets were again pelleted by centrifugation at 800× *g* for 15 min and the pellet was resuspended in either PBS or a HEPES-buffered Tyrode’s solution (137 mM NaCl, 12 mM NaHCO_3_, 2.8 mM KCl, 0.4 mM Na_2_HPO_4_, 1 mM MgCl_2_, 10 mM HEPES, 5.5 mM glucose, 2 mM CaCl_2_, pH 7.4). Platelet density was determined using a hemocytometer and adjusted to the appropriate concentration required for subsequent experiments.

### Cell culture

MDA PCa 2b cells were obtained from American Type Tissue Collection (ATCC, Manassas, VA) and maintained in Kaighn's Modification of Ham's F-12 medium supplemented with 20% FBS, 25 ng/ml cholera toxin (Sigma-Aldrich, St. Louis, MO), 10 ng/ml mouse Epidermal Growth Factor (Corning), 5 mM phosphoethanolamine (Sigma-Aldrich), 100 pg/ml hydrocortisone (Sigma-Aldrich), 45 nM sodium selenite (Sigma-Aldrich), 5 ug/ml human recombinant insulin (Life Technologies), and 1% penicillin–streptomycin. PC-3 cells were obtained from ATCC and maintained in ATCC-formulated F-K12 medium supplemented with 10% FBS. RC77 T/E and RC77 N/E cells were a generous gift from Johng Rhim^23^ and maintained in Keratinocyte-SFM medium (Gibco) supplemented with epidermal growth factor and bovine pituitary extract. All cell lines were maintained at 37 °C and 5% CO_2_.

### RNA isolation, RNA-Seq and transcriptome analysis

Information regarding the RNA-Seq data of platelets from healthy volunteers, including exclusion criteria, demographic data, and sample processing can be found in our previous publication^24^. RNA from PCa cell lines MDA PCa 2b and RC77 T/E was isolated using TRIzol extraction (Invitrogen, Waltham, MA), isopropanol-precipitated with GlycoBlue (Fisher Scientific, Pittsburgh, PA) and quality assessed using an Agilent 2100 Bioanalyzer system where RIN scores of all isolated RNA were ≥ 9.0. The resulting RNA were subjected to RNA-Seq and quality control as previously described^24^. Additional PCa cell line RNA-Seq data were obtained from GEO accession GSE110903 for transcriptome analysis.

A gene was defined as expressed if it met two criteria: (1) expression in at least 50% of the sequenced samples, and (2) average expression across all sequenced samples was at least 1 transcript per million (TPM). RNA-Seq data were subjected to gene ontology and pathway analyses using the Ingenuity Pathway Analysis (IPA, RRID:SCR_008653) computational tool (Qiagen, Germantown, MD) as previously described^24^.

### Light transmission aggregometry (LTA) of platelets

Aggregation was tested using a Model 490 4 + Four Channel Optical Aggregometer (Chronolog, Havertown, PA) according to manufacturer’s protocol. Platelet rich plasma (PRP) was collected from whole blood by centrifugation at 170× *g* for 15 min. Platelet poor plasma was collected from whole blood by centrifugation at 1500× *g* for 15 min. Platelets were stimulated with collagen (2 μg/ml), ADP (10 μM), epinephrine (10 μM), or thrombin (0.5 units/ml) for 10 min and maximum aggregation was measured.

### Invasion and caspase3/7 activity in PCa cell lines following stimulation with platelets

Invasion assays were performed using Corning BioCoat™ Matrigel^®^ Invasion Chambers with 8.0 µm PET Membrane (Thomas Scientific, Swedesboro, NJ) per manufacturer’s instructions. PCa cells (RC77 T/E or MDA PCa 2b) at 5 × 10^4^ were combined without or with platelets (at a 1:10 to 1:1000 ratio of cancer cells to platelets) in 200 µL of culture medium containing 0.1% FBS and seeded onto the top Matrigel invasion chamber, while 750 µL of medium with 20% FBS was added to bottom chamber. The PCa cell-platelet coculture was incubated for a total of 48 h. PCa cells invading through Matrigel and residing on the bottom side of the PET membrane were fixed, stained (nuclear and cytoplasm) with Diff Stain kit (IMEB Inc., San Marcos, CA), and counted under light microscope.

Apoptotic activity was measured using the Caspase-Glo 3/7 Assay (Promega) according to manufacturer’s protocol. A total of 20,000 MDA PCa 2b or RC77 T/E cells were seeded into a 96-well plate with platelets at a cell:platelet ratio of 1:10, 1:100, 1:500, or 1:1000. PCa cells alone (20,000 cells) and 20,000,000 platelets alone were also seeded into wells as controls. After incubation for 24 h at 37 °C, plates were gently washed with PBS and allowed to equilibrate to room temperature before a 1-h incubation with the Caspase-Glo reagent. Luminescence was measured using a Varioskan Flash Plate Reader (Thermo Fisher Scientific, Waltham, MA).

### Immunocytochemistry

MDA PCa 2b or RC77 T/E cells were incubated with washed platelets (at a ratio of 1:500 cancer cells to platelets) for 15 min at 37 °C while rocking before being transferred to a 4-chamber slide and incubating over night at 37 °C. The ratio 1:500 was chosen as it represented the lowest concentration of platelets that elicited maximal response in both invasion and caspase assays. Slides were fixed using 4% PFA, permeabilized with 0.5% Triton-X, and blocked with 1% BSA in PBS. Staining was carried out successively with a mouse antibody against CD41a (Cat # 14-0419-82, RRID: AB_467236), secondary Alexa Flour 546 (Cat # A-11030, RRID: AB_2534089) conjugated antibody specific to mouse IgG, guinea pig antibody recognizing the P2Y12 receptor (Cat # PA5-111827, RRID: AB_2857236), and finally secondary Alexa Flour 647 (Cat # A32733, RRID: AB_2633282) conjugated antibody against guinea pig IgG. All antibodies were purchased through Thermo Fisher Scientific (Waltham, MA). Each antibody was incubated for 1 h at room temperature, and three five-minute washes were performed between each incubation. After the final wash, the chamber barriers were removed and SlowFade™ Diamond Antifade Mountant with DAPI (Thermo Fisher Scientific, Waltham, MA, Cat #S36968) was used to mount the coverslip. All images were taken using a Leica DMi8 Inverted Fluorescent Microscope at 40× magnification. The identity of images was blinded and randomized during counting of PCa cell-platelet interactions. An interaction was defined as a platelet that is juxtaposed/bordering with a PCa cell. Counting was performed independently by at least two individuals and the mean count was calculated.

### Flow cytometry of activated platelets

Platelet activation was quantitated by surface expression of CD62P and activated integrin α_IIb_β_3_ following stimulation with either 0.5 units/ml thrombin for 5 min or PCa cells at the indicated cell:platelet ratios for 24 h. Briefly, 100 μl of activated platelet suspensions were fixed with 2% paraformaldehyde (PFA) and subsequently stained with an Alexa Flour 647 conjugated antibody recognizing CD61 (BioLegend, San Diego, CA, Cat #336407, RRID: AB_2128751) and a fluorescein isothiocyanate (FITC) conjugated antibody specific for CD62P (P-selectin antibody; BD Biosciences, San Jose, CA, Cat #555523, RRID: AB_395909), or stained directly (no fixative) with a FITC conjugated antibody recognizing PAC-1 (activated α_IIb_β_3_; BD Biosciences, San Jose, CA, Cat #340507, RRID: AB_2230769). Platelets were initially gated on the expression of platelet marker CD61 and then subsequently analyzed for expression of CD62P or activated α_IIb_β_3_ by flow cytometry (Accuri C6 Plus, BD Biosciences, San Jose, CA).

### PCa cell-stimulated calcium mobilization in platelets

PRP was obtained from whole blood samples by centrifugation at 170× *g* for 15 min with the centrifuge brake off and platelet count was adjusted to 300,000,000/ml. Preloading of platelets with Fluo 3-AM (Thermo Fisher Scientific) was accomplished by pretreating PRP with Fluo 3-AM at a final concentration of 5 µM at 37 °C for 30–45 min. PGE1 (250 nM final concentration) was added to the PRP mixture prior to centrifugation at 1500× *g* for 15 min with the brake on. The resulting pellet of washed platelets containing Fluo 3-AM was gently resuspended in HEPES-Tyrode’s buffer without calcium at a concentration of 300,000,000 platelets/ml. Washed pellets were added into individual wells of a 96-well plate in the presence of CaCl_2_ (2 mM final concentration) plus control IgG (5 µg/ml final concentration) or neutralizing antibody (5 µg/ml final concentration). Prior to adding PCa cells, baseline fluorescence of the plate was read (excitation: 488 nm, emission: 526 nm) every 30 s for 5 min using a Varioskan Flash Plate Reader (Thermo Fisher Scientific). The plate was then removed from the plate reader and 10 µL of PBS containing the appropriate amount of MDA PCa 2b cells (at a final ratio of 1:500, 1:1000, or 1:10,000 cancer cells to platelets) was added to each well. The plate was returned to the plate reader and PCa-stimulated fluorescence in platelets was recorded every minute for the first 5 min, followed by every 5 min for a total of 2 h.

### Transmission electron microscopy

MDA PCa 2b or RC77 T/E cells were incubated with washed platelets at a ratio of 1:500 cancer cells to platelets in culture medium for 12 h at 37 °C with gentle rocking. The PCa cell-platelet mixture was pelleted by centrifugation at 1000× *g* for 15 min at 37 °C. Cell-platelet pellets were fixed in 2.5% glutaraldehyde (Electron Microscopy Sciences), 1% paraformaldehyde in 0.12 M sodium cacodylate buffer (Electron Microscopy Sciences) for 1 h at room temperature. The fixed cell-platelet pellet was then enrobed in 4% agarose to keep the pellets together through processing. Briefly, pellets were fixed for 1 h in 1% osmium tetroxide (Electron Microscopy Sciences) followed by *en bloc* staining overnight in 1% aqueous uranyl acetate. The pellets were then dehydrated through a series of ethyl alcohol/deionized water solutions and propylene oxide before infiltration with Embed 812 epoxy resin. Blocks were cured for 48 h at 60 °C. Polymerized blocks were trimmed, and 95-nm-ultrathin sections were cut with a diamond knife on a Leica Ultramicut EM UC7 and transferred onto 200 mesh copper grids. Sections were counterstained with 1% uranyl acetate for 10 min and lead citrate for 2 min. Samples were imaged with a FEI Talos F200X transmission electron microscope (Thermo Fisher Scientific, Waltham, MA) operating at an accelerating voltage of 80 kV equipped with a Ceta™ 16 M camera.

### Identifying signaling axis partners responsible for PCa cell-platelet stimulated events

To assess and identify signaling axis partners responsible for PCa-platelet stimulated events, neutralizing antibodies, cytokines or small molecule inhibitors were included in functional assays measuring calcium mobilization, invasion and caspase 3/7 activity. Antibodies were employed at a final concentration of 5 µg/ml to neutralize IL32 (Abcam, Cat #ab37158), CCL3L1 (Abcam, Cat #ab259372), PAC-1 (BD Biosciences, San Jose, CA, Cat #340535), FN1 (Sigma-Aldrich, Cat #F3648, RRID:AB_476976), ADGRE5 or CD97 (Abcam, Cambridge, MA, Cat #ab108368, RRID:AB_10865208) and CD55 (R&D Systems, Cat#MAB2009, RRID:AB_2075961). Cytokines included IL32 (50 nM final concentration; R&D Systems, Minneapolis, MN, Cat #3040-IL) and CCL3L1 (10 nM final concentration; Abcam, Cambridge, MA, Cat #ab50065). Small molecule inhibitors (10 µM final concentration) included the LPAR antagonist H2L 5765834 (Bio-Techne, Minneapolis, MN, Cat #4870) and pan-EPH receptor antagonist UniPR1331 (Aobious, Gloucester, MA, Cat # AOB11775).

### Statistical analysis

Functional data were analyzed by Student’s t-test at *P* < 0.05, or ANOVA and Dunnett’s or Tukey’s *post-hoc* test at *P* < 0.05. Survival plots were analyzed by logrank test at *P* < 0.05.

## Results

### PCa cells directly interact with platelets through developing microvilli-like structures

**Figure 1:**
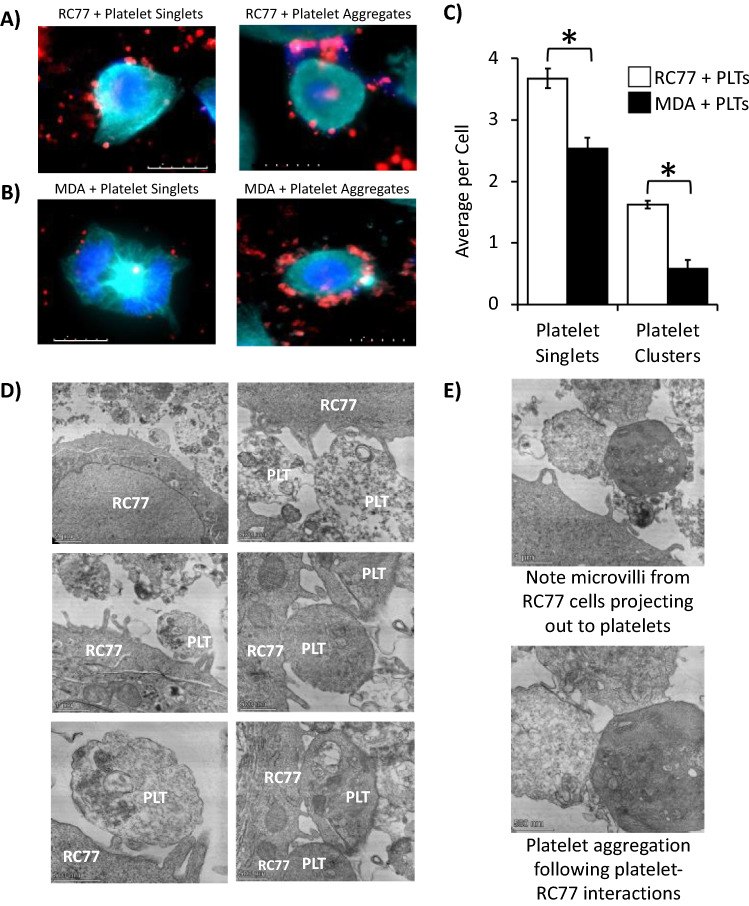
PCa cells physically interact with platelets. Representative fluorescent images of (**A**) RC77 T/E and (**B**) MDA PCa 2b cells incubated with platelets at a cell:platelet ratio = 1:500. Cells were stained with antibody for the P2Y12 receptor (cyan) and DAPI for DNA (blue). Platelets were stained with an antibody for CD41a (red). Inset depicts scale bar in 5 µm intervals. (**C**) Quantification of platelet singlets and clusters/aggregates interacting with RC77 T/E (n = 109 imaged cells incubated with platelets at a cell:platelet ratio = 1:500) and MDA PCa 2b (n = 127 imaged cells incubated with platelets at a cell:platelet ratio = 1:500). Bar graphs represent the mean ± SEM; Student’s *t*-test, *P < 0.05. (**D**) Representative transmission electron micrograph (TEM) images depicting physical interactions between RC77 T/E cells and platelets. (**E**) Magnified image of microvilli-like structures projecting from RC77 T/E cell toward platelet aggregates (top panel) and representative image of platelet-platelet focal adhesions indicative of PCa cell-stimulated platelet aggregation (bottom panel).

To assess the nature of platelet-prostate cancer (PCa) cell interactions in vitro, we incubated either RC77 T/E or MDA PCa 2b cells with platelets at a PCa cell:platelet ratio of 1:500 for 24 h. Immunofluorescent staining revealed platelets in an apparent interaction with RC77 T/E and MDA PCa 2b cells, either as individual platelets (or singlets) or as clusters of two or more aggregated platelets (Fig. 1A,B). The latter event is highly suggestive that PCa cell-platelet interactions promote platelet activation/aggregation. The vast majority of PCa cells (78% for MDA PCa 2b and 99% for RC77 T/E cells) interacted with platelets. Each individual RC77 T/E cell interacted on average with 3.7 individual platelets and 1.6 platelet clusters, while each individual MDA PCa 2b cell interacted with 2.5 individual platelets and 0.6 platelet clusters (Fig. 1C).

Transmission electron microscopy (TEM) revealed RC77 T/E cells with microvilli-like protrusions projecting outwards and in apposition with individual platelets (Fig. 1D, left panels). Furthermore, the microvilli-like protrusions emanating from RC77 T/E cells were also found in direct contact with the platelet aggregates (Fig. 1D, right panels; and 1E). The platelets within the aggregates exhibited platelet-to-platelet focal adhesion (Fig. 1E). However, the platelets comprising these aggregates retained their original round discoid shape and did not undergo profound shape change (such as spreading and numerous filopodia extensions) typically associated with platelet activation following agonist stimulation (such as collagen-stimulated platelet aggregation). Noteworthy, finger-like protrusions were less numerous and prominent on the surface of RC77 T/E cells in the absence of platelet incubation (data not shown). In summary, these micrographs reveal platelet-to-PCa cell physical interactions with apparent ‘atypical’ platelet activation and aggregation; observations not previously described nor demonstrated at this scale of resolution.

### PCa cells activate platelets

**Figure 2:**
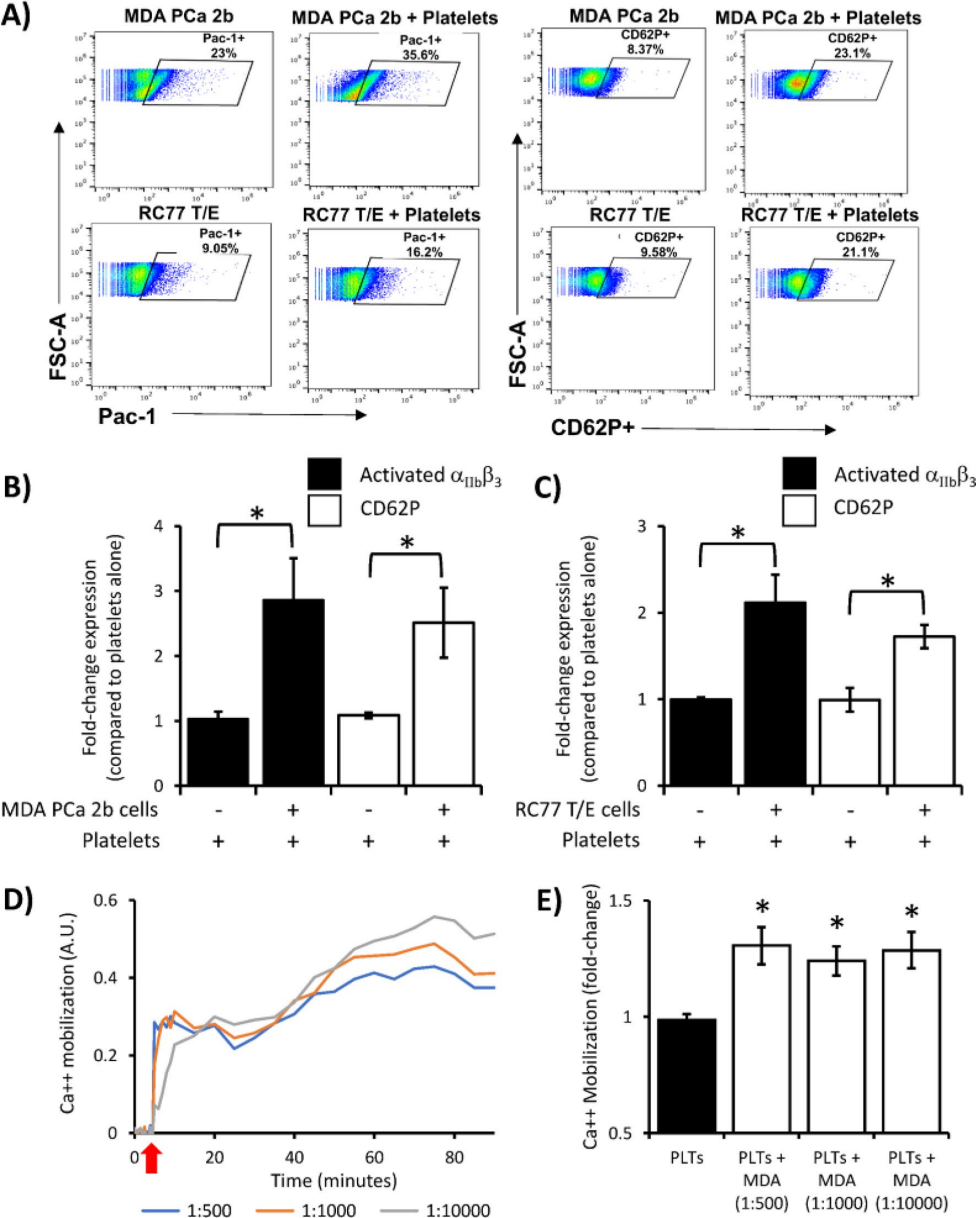
PCa cell lines MDA PCa 2b and RC77 T/E activate platelets. (**A**) Representative flow cytometry dot plots for surface expression of two platelet activation markers, activated integrin α_IIb_β_3_ (PAC-1) and P-selectin (CD62P). Quantification of flow cytometry of platelets incubated with (**B**) MDA PCa 2b or (**C**) RC77 T/E cells for 24 h at a cell:platelet ratio of 1:1000. Data presented as the mean ± SEM of n = 5–6 independent determinations and analyzed by Student’s *t*-test, *P < 0.05. (**D**) Representative plot of calcium mobilization in platelets over 90 min following addition of MDA PCa 2b (MDA) cells at the indicated cell:platelet ratios. Red arrow indicates addition of MDA cells. A.U., arbitrary units. (**E**) Data presented as the mean ± SEM of n = 4–7 independent determinations at the indicated MDA cell:platelet (PLT) ratios and analyzed by ANOVA and Dunnett’s or Tukey’s *post-hoc* test, *P < 0.05.

To test the hypothesis that platelet interaction with PCa cells leads to platelet activation, we used flow cytometry to measure expression of CD62P (also known as P-selectin) and activated integrin α_IIb_β_3_ on the surface of platelets. Increased surface expression of these two proteins is indicative of platelet activation following incubation of platelets with classic agonists (such as thrombin, ADP and collagen)^25^. Following 24-h incubation of platelets with MDA PCa 2b cells, at a cell:platelet ratio of 1:1000, the number of CD62P-positive and activated α_IIb_β_3_-positive platelets significantly increased 2.8- and 2.5-fold over baseline, respectively (Fig. 2A,B). When platelets were incubated with RC77 T/E cells for the same amount of time at a cell:platelet ratio of 1:1000, the number of CD62P-positive and activated α_IIb_β_3_-positive platelets likewise increased 2.1- and 1.7-fold, respectively (Fig. 2A,C).

Platelet activation by PCa cells was also associated with calcium mobilization. We preloaded platelets with a calcium-sensing fluorescent dye and measured calcium mobilization following PCa cell addition for 90 min (Fig. 2D). Calcium mobilization in platelets was significantly increased ~ 1.4-fold above baseline following incubation with MDA PCa 2b cells at cell:platelet ratios of 1:500, 1:1000 and 1:10,000 (Fig. 2E). This observation, in addition to the significant increase in activated platelet surface markers α_IIb_β_3_ and CD62P following incubation of platelets with PCa cells, suggest that PCa cells can facilitate platelet activation.

### Platelets increase invasion and decrease caspase activity in PCa cells

**Figure 3:**
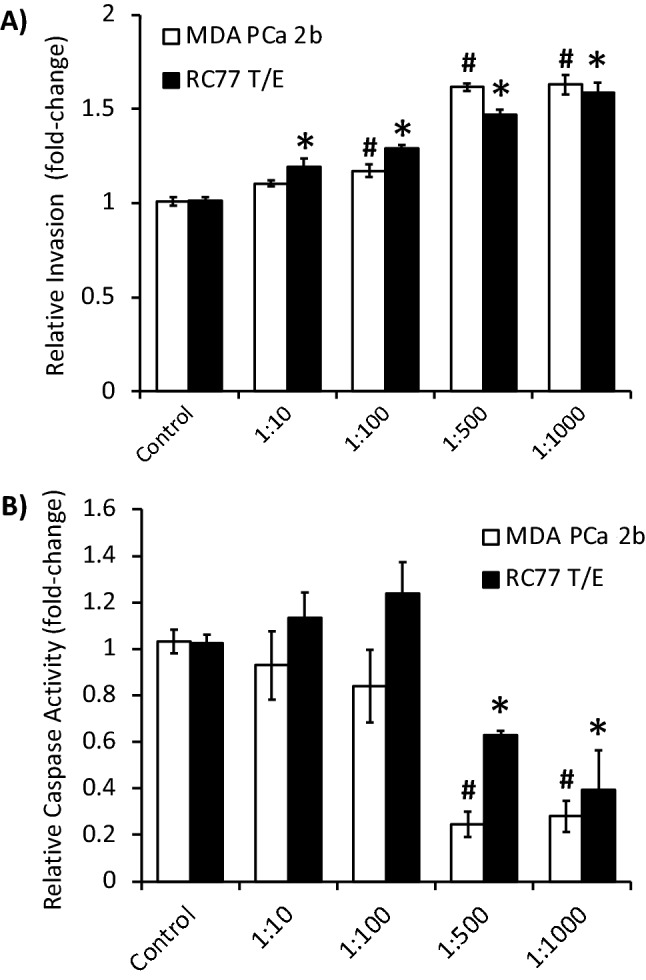
Platelets increase invasion and decrease caspase activity in PCa cells. (**A**) Platelet-stimulated PCa cell invasion. Invasion was determined by Matrigel assay. Data presented as the mean ± SEM of n = 4–7 independent determinations at the indicated PCa cell:platelet ratios and analyzed by ANOVA and Dunnett’s *post-hoc* test. * or ^#^, *P* < 0.05; significantly different from corresponding control. (**B**) Platelet-induced PCa cell apoptotic resistance. Apoptotic resistance was determined by caspase 3/7 activity assay. Data presented as the mean ± SEM of n = 4–9 independent determinations at the indicated PCa cell:platelet ratios and analyzed by ANOVA and Dunnett’s *post-hoc* test. * or ^#^, *P* < 0.05; significantly different from corresponding control.

Given that MDA PCa 2b and RC77 T/E cells can activate platelets, we next tested whether platelets could reciprocally activate these two PCa cell lines. Consequently, the ability of platelets to promote PCa cell invasion in a Matrigel assay was investigated. Invasion of MDA PCa 2b cells was significantly enhanced in a dose-dependent manner when incubated with increasing amounts of platelets at a PCa cell:platelet ratio of 1:10 to 1:1000 (Fig. 3A). A maximal 1.6-fold increase in invasion was observed at an MDA PCa 2b cell:platelet ratio of 1:500. Similar dose-dependent results were observed upon incubation of platelets with RC77 T/E cells (Fig. 3A).

Next, we tested the effect of platelets on apoptosis of PCa cell lines by measuring caspase 3/7 activity. Caspase 3/7 activity in both MDA PCa 2b and RC77 T/E cells was significantly reduced in a dose-dependent manner following incubation with increasing amounts of platelets at ratios ranging from 1:10 to 1:1000 (Fig. 3B). A maximal fourfold decrease in caspase 3/7 activity was observed in MDA PCa 2b cells at a cell:platelet ratio of 1:500. We also tested the PCa cell line PC-3, which is androgen receptor-negative in contrast to the androgen receptor-positive and/or androgen-sensitive MDA PCa 2b and RC77 T/E cells^23,26^. Platelets were capable of stimulating invasion of PC-3 cells in a manner analogous to MDA PCa 2b and RC77 T/E cells, but not apoptotic resistance (Supplementary Fig. S1A). These findings suggested potential androgen receptor dependency of platelet-stimulated apoptotic resistance and/or genetic differences in cell lines. Subsequently, we tested platelet stimulation of MDA PCa 2b and RC77 T/E cells in the presence of androgen receptor blockade with 20 µM bicalutamide. Platelet-stimulated invasion and apoptotic resistance in MDA PCa 2b and RC77 T/E cells were insensitive to bicalutamide (Supplementary Fig. S1B,C). Taken together these findings revealed that platelets have both pro-invasive and anti-apoptotic effects on PCa cell lines that were independent of the androgen receptor.

The effects of platelets on the prostate epithelial cell line RC77 N/E (derived from non-cancerous prostate tissue^23^) and vice versa were also assessed. Co-incubation of RC77 N/E cells with platelets at a ratio of 1:1000 did not lead to an increase in cell invasion (1.09 ± 0.04-fold change compared to cells alone; N = 3, *P* > 0.05), nor a reciprocal increase in platelet calcium mobilization (0.97 ± 0.11-fold change compared to platelets alone; N = 3, *P* > 0.05).

### Cataloguing candidate signaling axis pairs participating in PCa cell-platelet interactions

**Figure 4:**
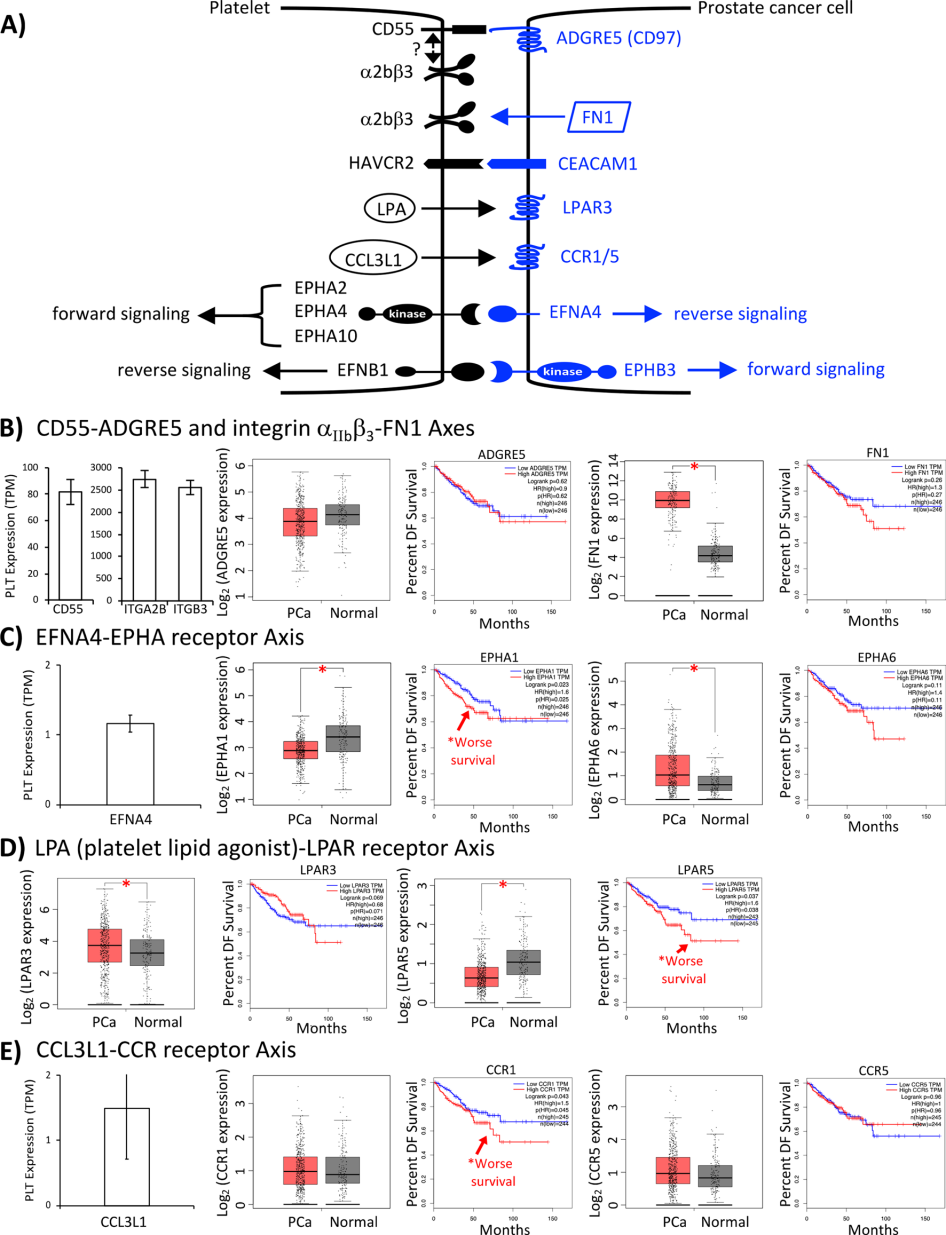
Candidate PCa cell-platelet signaling axis partners. (**A**) Schematic representation of potential signaling axes mediating PCa cell-platelet interactions. (**B**–**E**) Bar graphs depict expression of platelet signaling partner and corresponding PCa signaling partner based on Garofano et al.^24^ and TCGA RNA-Seq data. Disease free (DF) survival plots are depicted for PCa signaling partners based on TCGA data. Signaling axes (platelet component-PCa component) displayed include (**B**) CD55-ADGRE5 and α_IIb_β_3_-FN1, (**C**) EFNA4-EPHA receptor, (**D**) LPA-LPAR receptor, and (**E**) CCL3L1-CCR receptor. TPM, transcript per million; *, Student’s *t*-test or logrank test, *P < 0.05.

PCa cell-platelet interactions were associated with reciprocal activation events (platelet activation and aggregation; PCa cell invasion and apoptotic resistance). Consequently, we sought to identify the surface receptor-ligand interactions mediating these activities. PCa surface proteins (receptors or ligands) that acted as signaling axis partners to corresponding platelet surface proteins (ligands or receptors) were inferred by searching RNA-Seq datasets from TCGA (https://www.cancer.gov/tcga) and Garofano et al.^24^, respectively. As depicted in Fig. 4A (and Supplementary Fig. S2), we identified multiple candidate signaling pairs that could potentially mediate the observed aggregatory and oncogenic events. These signaling axis pairings were broadly divided into the following categories: (1) integrin-integrin ligand (e.g. integrin α_IIb_β_3_-fibronectin1 (α_IIb_β_3_-FN1) and integrin α_6_β_1_-laminin), (2) ephrin-EPH receptor (ephrin A4-Eph receptor A1 (EFNA4-EPHA1)), (3) immune checkpoint receptor-ligand (hepatitis A virus cellular receptor 2-carcinoembryonic antigen related cell adhesion molecule 1 (HAVCR2-CEACAM1)), and (4) miscellaneous ligand-receptor interactions (chemokine ligand 3 like 1-CC chemokine receptor type 1 (CCL3L1-CCR1) and CD55-ADGRE5). Many of the candidate signaling axis pairings were comprised of an axis partner on the PCa side that was differentially expressed in PCa versus normal prostate. For example, *FN1* and *ERBB3* were significantly overexpressed in PCa (Fig. 4B–E, Supplementary Fig. S2). Moreover, numerous examples were found where high expression of the PCa axis partner was associated with worse patient survival. Examples included cytokine *IL32*, *DLL's* (ligands for Notch), *CCR's* (chemokine receptors for platelet-derived *CCL3L1*), and *LPAR's* (receptors for platelet-derived LPA) (Fig. 4B–E, Supplementary Fig. S2). These general findings derived from TCGA were validated by analyzing the Taylor et al. dataset^27^, which comprises a separate cohort of PCa specimens interrogated with the Affymetrix Human Exon 1.0 ST array. A comparison of Supplementary Fig. S3 (based on analysis of the Taylor et al. Affymetrix arrays) with Fig. 4 and Supplementary Fig. S2 (based on analyzing the TCGA RNA-Seq) revealed good overall concordance between the two datasets.

### Identifying the signaling axis pair participating in PCa cell-stimulated platelet activation

**Figure 5:**
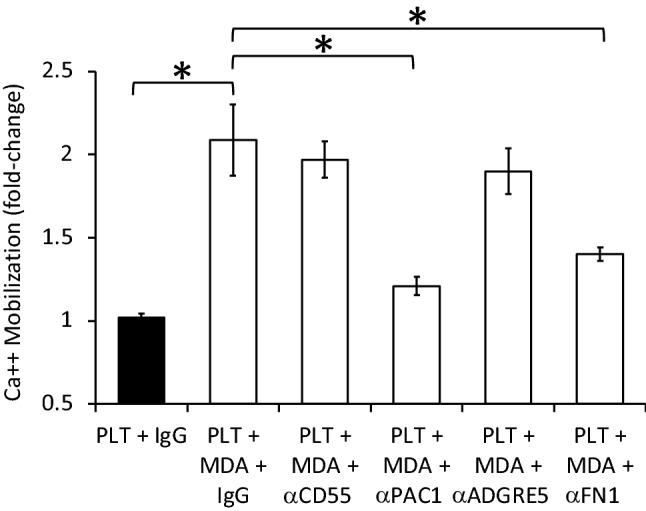
α_IIb_β_3_-FN1 signaling axis mediates PCa cell-stimulated calcium mobilization in PLTs. MDA PCa 2b (MDA)-stimulated calcium mobilization in platelets (PLT) was examined in the presence of IgG isotype control (5 µg/ml) or indicated blocking antibody (α; 5 µg/ml). Cell:PLT ratio was 1:1000. Data presented as the mean ± SEM of n = 6–9 independent determinations and analyzed by ANOVA and Tukey *post-hoc* test, *P < 0.05.

Antibody neutralization experiments (5 µg/ml for all antibodies tested) were performed to validate the signaling axis governing PCa cell-stimulated platelet activation. The first signaling protein on the surface of platelets to be targeted for neutralization was integrin α_IIb_β_3_ given its prominent role in platelet aggregation following stimulation of platelets with the classic platelet agonists thrombin, ADP and collagen^25^. Moreover, the genes *ITGA2B* and *ITGB3* that encode integrin α_IIb_β_3_ were the most highly transcribed of the candidate signaling axis partners (see Fig. 4 and Supplementary Fig. S2; Supplementary Table S1). Incubation of MDA PCa 2b cells with washed platelets at a concentration ratio of 1:500 (PCa cell:platelet) in the presence of IgG isotype control (5 µg/ml) resulted in a twofold increase in platelet-specific calcium mobilization compared to platelets incubated with 5 µg/ml IgG control alone (Fig. 5). The MDA PCa 2b cell-stimulated calcium mobilization was significantly abrogated by the neutralizing antibody PAC-1 that targets the activated form of integrin α_IIb_β_3_ (Fig. 5). Next, a reciprocal neutralization experiment was performed by targeting the G-protein coupled receptor ADGRE5 (also known as CD97), a known signaling axis partner of integrin α_IIb_β_3_ albeit ADRE5 does not bind directly to the integrin^22^. *ADGRE5* was found to be moderately expressed in the PCa cell lines MDA PCa 2b (TPM = 29) and RC77 T/E (TPM = 23) based on our RNA-Seq results (Supplementary Table S1), as well as PCa specimens based on TCGA RNA-Seq data (Fig. 4A,B). Preincubation of MDA PCa 2b cells with a neutralizing antibody against ADGRE5 was unable to reverse MDA PCa 2b cell-stimulated calcium mobilization in platelets (Fig. 5). We then targeted CD55, the known transmembrane ligand of ADGRE5^28^ that was found to be moderately expressed in platelets (RNA-Seq mean TPM expression value = 82 ± 10; N = 43 volunteers^24^), for targeted neutralization. As anticipated based on the results of ADGRE5 neutralization, reciprocal neutralization of CD55 did not abrogate MDA PCa 2b cell-stimulated calcium mobilization in platelets (Fig. 5). Following these negative findings, an alternative α_IIb_β_3_ signaling partner was selected for targeted neutralization (Fig. 4A,B). FN1 represented an ideal candidate given its high expression in MDA PCa 2b (TPM = 873; Supplementary Table S1) and RC77 T/E cells (TPM = 1078), as well as being a known ligand activator of α_IIb_β_3_^25^ and secreted protein of PCa^29^. Neutralization of FN1 resulted in a significant disruption of MDA PCa 2b-stimulated calcium mobilization in platelets (Fig. 5). It should be noted that platelets do not express appreciable *FN1* based on our prior RNA-Seq analysis in platelets (mean TPM = 0.53 ± 0.11; N = 43 volunteers)^24^.

We have previously demonstrated that platelet-secreted cytokines, IL32 and CCL3L1, participate in collagen-stimulated platelet activation and aggregation^24^. Hence, conditioned medium derived from platelets cultured alone, MDA PCa 2b cells cultured alone, or platelets + MDA PCa 2b cells cocultured for 90 min was added to platelets. None of these conditions was sufficient to stimulate calcium mobilization, indicating that the continued presence of PCa cells was necessary for PCa cell-stimulated platelet activation (Supplementary Fig. S4A).

### Identifying the signaling axis pairs participating in platelet-stimulated PCa cell invasion

**Figure 6:**
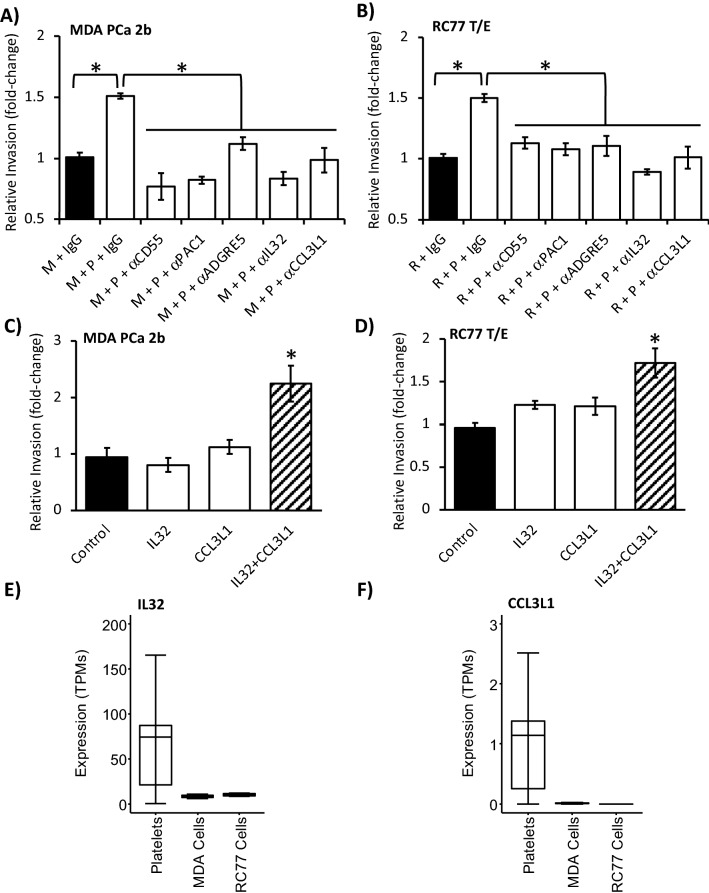
CD55/α_IIb_β_3_-ADRE5, CCL3L1-CCR and IL32 signaling axes participate in PLT-stimulated PCa cell invasion. (**A**, **B**) Platelet (P)-stimulated MDA PCa 2b (M) and RC77 T/E (R) invasion was examined in the presence of IgG isotype control (5 µg/ml) or the indicated blocking antibody (α; 5 µg/ml). Cell:platelet ratio was 1:1000. Data presented as the mean ± SEM of n = 8–10 independent determinations and analyzed by ANOVA and Tukey *post-hoc* test, *P < 0.05. (**C**, **D**) PCa cell invasion was measured following exogenous administration of 50 nM IL32 alone, 10 nM CCL3L1 alone or the combination of IL32 and CCL3L1. Data presented as the mean ± SEM of n = 3 independent determinations and analyzed by ANOVA and Dunnett’s *post-hoc* test, *, *P* < 0.05; significantly different from control. (**E**, **F**) IL32 and CCL3L1 are expressed in platelets, but absent or nearly absent in MDA PCa 2b and RC77 T/E PCa cell lines based on RNA-Seq analysis. The expression levels are given as transcripts per million (TPM) and presented as Box-and-Whiskers plots. Box: top quantile, mean and bottom quantile. Whiskers: top extreme (90 percentile of the dataset) and bottom extreme (10 percentile of the dataset). Platelet values derived from RNA-Seq of n = 44 healthy volunteers^24^. PCa cell line values derived from RNA-Seq (GEO accessions GSE213223 (current study) and GSE110903).

Analogous antibody neutralization experiments were performed to ascertain the signaling axis pair(s) responsible for platelet-stimulated PCa cell invasion. Incubation of either MDA PCa 2b or RC77 T/E cells with washed platelets at a concentration ratio of 1:1000 (PCa cell:platelet) in the presence of IgG isotype control resulted in a ~ 1.5-fold increase in PCa cell invasion compared to PCa cells incubated with IgG control alone (Fig. 6A,B). The platelet-stimulated invasion was significantly abrogated upon preincubation of MDA PCa 2b or RC77 T/E cells with a neutralizing antibody targeting ADGRE5 (Fig. 6A,B). In a reciprocal manner, neutralization of CD55 on platelets reversed platelet-stimulated invasion (Fig. 6A,B). Moreover, neutralization of α_IIb_β_3_ (indirect signaling axis partner with ADGRE5^22^), likewise inhibited platelet-stimulated PCa invasion (Fig. 6A,B). These findings were particularly interesting as neutralization of the α_IIb_β_3_-FN1, but not the CD55-ADRE5, axis reversed PCa cell-stimulated calcium mobilization in platelets (compare Fig. 5 with Fig. 6A,B).

Conditioned medium from platelet-cancer cell cocultures has previously been shown to have high MMP-9 activity and stimulate cancer cell invasion^30–32^. Consequently, conditioned medium derived from platelets cultured alone, PCa cells cultured alone, or platelets + PCa cells cocultured for 48 h was added to PCa cells. Only the latter promoted PCa cell invasion (Supplementary Fig. S4B), indicating that the continued presence of platelets was not a requirement for stimulated PCa cell invasion. Next, we tested whether platelet-secreted cytokines IL32 and CCL3L1^24^ participated in platelet-stimulated PCa cell invasion. Neutralization of either cytokine significantly reversed the ability of platelets to increase invasiveness of MDA PCa 2b and RC77 T/E cells at a cell:platelet ratio of 1:1000 (Fig. 6A,B; compare IgG control to neutralization antibody). Subsequently, we directly added the cytokine IL32 and/or CCL3L1 to PCa cell lines and measured invasion. Individual treatment with either 50 nM IL32 or 10 nM CCL3L1 had no significant effect on MDA PCa 2b and RC77 T/E invasion (note that platelet incubation was excluded in these experiments), while the combination of both cytokines significantly increased PCa cell invasion by ~ twofold over saline vehicle control (Fig. 6C,D). Taken together, these experiments demonstrate that the platelet cytokines IL32 and CCL3L1, shown previously to participate in platelet activation^24^, also play a role in platelet-stimulated PCa cell invasion. It should be noted that these cytokines were not expressed to weakly expressed in MDA PCa 2b and RC77 T/E cells based on RNA-Seq (Fig. 6E,F).

### Identifying the signaling axis pairs participating in platelet-stimulated PCa cell apoptotic resistance

**Figure 7:**
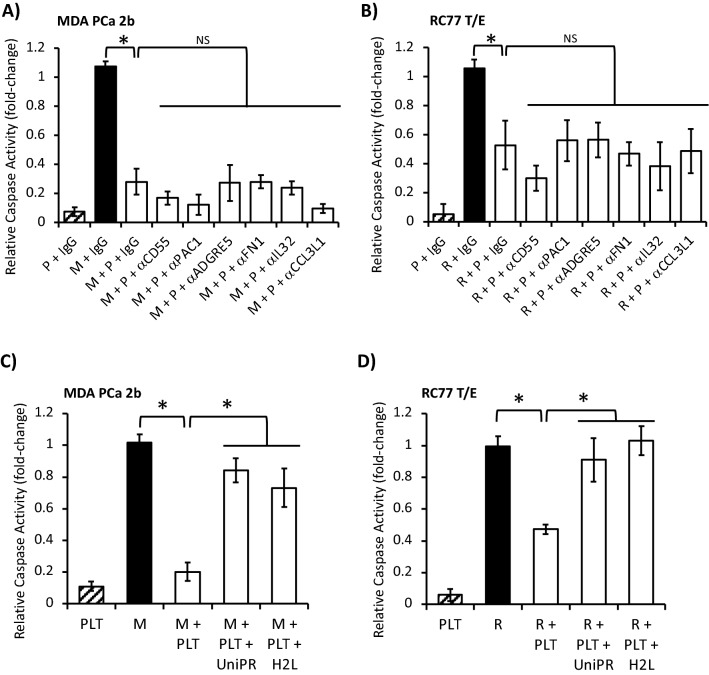
Ephrin-EPH receptor and LPA-LPAR signaling axes participate in platelet-induced PCa cell apoptotic resistance. (**A**, **B**) Platelet (P)-induced MDA PCa 2b (M) and RC77 T/E (R) apoptotic resistance was examined in the presence of IgG isotype control (5 µg/ml) or the indicated blocking antibodies (α; 5 µg/ml). Cell:platelet ratio was 1:1000. Data presented as the mean ± SEM of n = 4–8 independent determinations and analyzed by ANOVA and Tukey *post-hoc* test, *P < 0.05. (**C**, **D**) Platelet-induced MDA PCa 2b and RC77 T/E apoptotic resistance was examined in the absence or presence of pan-receptor subtype inhibitors UniPR1331 (UniPR; 10 µM) and H2L 5765834 (H2L; 10 µM) to uncouple the ephrin-EPH receptor and LPA-LPAR axes, respectively. Cell:platelet ratio was 1:1000. Control indicates PCa cells in the absence of platelets and treated with vehicle. PLT indicates addition of platelets to PCa cells. Data presented as the mean ± SEM of n = 3–6 independent determinations and analyzed by ANOVA and Tukey *post-hoc* test, *P < 0.05.

Lastly, the signaling axis responsible for platelet-induced apoptotic resistance in MDA PCa 2b and RC77 T/E cells was assessed. Neutralization experiments targeting α_IIb_β_3_, CD55, ADRE5 and FN1 were ineffective, indicating that neither the CD55/α_IIb_β_3_-ADRE5 axis promoting invasion nor the α_IIb_β_3_-FN1 axis mediating calcium mobilization contributed to apoptotic resistance (Fig. 7A,B). Likewise, neutralization of IL32 and CCL3L1 (components found to be important for platelet-stimulated PCa cell invasion) was ineffective in abrogating platelet-induced inhibition of caspase 3/7 activity in PCa cell lines (Fig. 7A,B). Next, we assessed alternative receptor-ligand pairings with the following criteria: (1) the PCa signaling component was differentially expressed in PCa compared to normal prostate and (2) high expression of the PCa signaling component was associated with worse patient survival. Two ligand-receptor pairs matching these criteria were the ephrin-EPH receptor and LPA-LPAR signaling axes (Fig. 4 and Supplementary Fig. S2). The repertoire of EPH receptors and LPARs expressed in MDA PCa 2b and RC77 T/E cells based on RNA-Seq (Supplementary Table S1) included *EPHA1* (MDA PCa 2b TPM = 10, RC77 T/E TPM = 25), *LPAR2* (MDA PCa 2b TPM = 11, RC77 T/E TPM = 12), *LPAR3* (MDA PCa 2b TPM = 49, RC77 T/E TPM = 20), *LPAR5* (MDA PCa 2b TPM = 1, RC77 T/E TPM = 12) and *LPAR6* (MDA PCa 2b TPM = 13, RC77 T/E TPM = 23). We employed the pan-receptor subtype inhibitors UniPR1331 (10 µM final) and H2L 5765834 (10 µM final) to uncouple the ephrin-EPH receptor and LPA-LPAR axes, respectively. Co-incubation of MDA PCa 2b with either UniPR1331 or H2L 5765834 was sufficient to substantially abrogate the fivefold decrease in caspase 3/7 activity induced by platelets (Fig. 7C). Similarly, co-incubation of RC77 T/E cells with either UniPR1331 or H2L 5765834 was sufficient to fully abrogate the twofold decrease in caspase 3/7 activity induced by platelets (Fig. 7D). These findings suggest that the ephrin-EPH receptor and LPA-LPAR signaling axes act cooperatively during platelet-induced apoptotic resistance in PCa cells.

Conditioned medium derived from platelets cultured alone, PCa cells cultured alone, or platelets + PCa cells cocultured for 24 h was added to PCa cells. None of these conditions was sufficient to stimulate apoptotic resistance (Supplementary Fig. S4C), indicating that the continued presence of platelets was necessary during platelet-stimulated PCa cell apoptotic resistance.

## Discussion

CTC and circulating platelet counts in patients with localized PCa average 107 per ml^33^ and 228 million per ml of blood^34^, respectively. For PCa patients with metastatic disease, CTC and circulating platelet counts average 116 per ml^33^ and 232 million per ml of blood^34^, respectively. Given these numbers, the CTC:platelet ratio in blood is estimated to be 1:2,000,000 in metastatic PCa. Our study demonstrates that platelets can stimulate invasion and promote apoptotic resistance in two PCa cell lines MDA PCa 2b and RC77 T/E at cell:platelet ratios of 1:10 and 1:500, respectively. Reciprocally, PCa cells stimulated platelet aggregation, calcium mobilization in platelets and induced platelet surface expression of P-selectin and α_IIb_β_3_ at comparable cell:platelet ratios. Taken together, our findings indicate that bidirectional signaling between PCa cells and platelets is exceedingly sensitive and occurs at platelet levels ~ 4,000 to 200,000-fold lower than normally encountered in the blood.

There is accumulating evidence that platelet-tumor cell interactions promote thrombus formation. The phenomenon of TCIPA has been observed with breast, colon, lung, osteosarcoma, pancreatic, ovarian, and prostate cancer cell lines^14,32,35–37^. The more common and conventional approach to measure TCIPA has been light transmission aggregometry, which does not provide qualitative information regarding the nature of platelet-cancer cell interactions. Our strategy of applying both fluorescence microscopy and TEM demonstrated that platelets can interact directly with PCa cells as platelet singlets as well as clusters of aggregated platelets. At the ultrastructural level, platelet singlets and clusters/aggregates contact PCa cells through microvilli-like structures emanating from the latter. We do not know at this time if microvilli-like formation is a prerequisite for TCIPA and/or platelet-stimulated events in PCa cells (such as invasion and apoptotic resistance). Notably, classic platelet agonists (ADP, thrombin and collagen) mediate platelet aggregation within seconds to minutes whereas TCIPA occurs on a scale of tens of minutes^14,32,35–37^. It is possible that platelet-tumor cell interactions promote microvilli formation, serving as a platform for the shedding of extracellular vesicles containing pro-aggregatory and pro-oncogenic signaling molecules and/or microRNAs^38^.

Classic platelet agonists will stimulate the transport of α-granules to the platelet surface resulting in the release of signaling molecules (such as hemostatic factors, growth factors, angiogenic factors and cytokines) and translocation of α_IIb_β_3_ and P-selectin onto the platelet surface^25^. Agonist stimulation also promotes activation, shape change and aggregation of platelets^25^. While all these agonist-mediated events are typically described as being calcium-dependent, calcium-independency has also been ascribed^39,40^. In a manner mimicking classic agonists, PCa cells have been demonstrated to stimulate platelets leading to secretion of ADP, ATP, LPA and TGF-β^22,41,42^. However, PCa cell-induced calcium mobilization from intracellular stores in platelets has not been explored to date, nor has this phenomenon been extensively investigated for other cancer types. We demonstrate that PCa cell stimulation of calcium mobilization in platelets occurred at cell:platelet incubation ratios ranging from 1:500 to 1:1000. Noteworthy, the 1.4-fold maximum increase in calcium mobilization induced by PCa cells was substantially lower than the 5–10 fold increase typically observed with the classic platelet agonists ADP, thrombin and collagen^43^. An earlier study reported that TCIPA can be initiated by androgen receptor-negative PCa cell lines DU145 and PC-3, but not androgen receptor-positive lines LNCaP and RWPE-1^44^. Our study, employing MDA PCa 2b and RC77 T/E PCa cells, demonstrates that these androgen receptor-positive lines^45,46^ can indeed initiate TCIPA. Given the significant, albeit modest, PCa cell-induced calcium mobilization in platelets, we also evaluated the ability of PCa cells to promote expression of integrin α_IIb_β_3_ and P-selectin on the surface of platelets. Increased surface expression of α_IIb_β_3_ and P-selectin is known to contribute to platelet aggregation^25^, representing a phenomenon that has not been adequately addressed in cancer cell-platelet interactions. Our findings demonstrate that incubation of platelets with PCa cells was sufficient to increase the number of integrin-positive and P-selectin-positive platelets by ~ 2–3 fold, albeit this value was substantially lower than the 100–150-fold increase obtained following incubation with classic platelet agonists such as thrombin (see Supplementary Fig. S5).

Platelet-stimulated metastatic programming has been noted in several cancer types, including breast, colon, melanoma and pancreatic cancer cell lines (see for example^32,47–49^. In PCa cells, platelets will induce EMT^2^, invasion^20,22^, and extravasation of PCa cells^2,22^. In addition, platelet-derived microparticles have been demonstrated to stimulate an angiogenic program in PCa cells, leading to angiogenic-related gene expression changes and increased expression of IL8, a known pro-angiogenic factor^50^. However, the ability of platelets to promote other hallmarks of cancer in PCa cells (such as proliferation, survival/anti-apoptosis, deregulated cellular energetics, etc.) remain ambiguous. This study now establishes that platelets can promote apoptotic resistance in MDA PCa 2b and RC77 T/E cells by decreasing caspase 3/7 activity. Our findings parallel other studies demonstrating the ability of platelets to inhibit cancer cell apoptosis. This includes a murine model of ovarian cancer^10^ and in vitro model of platelet-stimulated suppression of cisplatin-induced caspase activity in non-small cell lung cancer cells^51^. Taken together, it seems likely that platelet-stimulated apoptotic resistance is a common theme across different cancer types.

We have previously ascertained that approximately 10% of the 13,415 expressed genes in platelets encode diverse transmembrane receptors, secreted ligands, transmembrane ligands, and immune-related surface molecules (i.e. checkpoint molecules)^24^. Many of these signaling molecules have the potential to mediate crosstalk with PCa cells (e.g. platelet-stimulated PCa cell invasion and apoptotic resistance, PCa cell-stimulated platelet activation and aggregation). Consequently, we were interested in identifying and functionally validating the corresponding signaling partners located on PCa cells. Of interest, an unusually high fraction (21 out of 26 or 81%; see Fig. 4 and Supplementary Fig. S2) of candidate partners was identified as differentially expressed in PCa compared to normal prostate and/or significantly associated with worse PCa survival outcome. Several of these candidates are now established by our study as likely participating in PCa-platelet crosstalk (i.e. FN1, CCR1 (receptor for CCL3L1), EPHA1, EPHA6, EFNA4, EPHB3, LPAR3, LPAR5).

In an earlier study, integrin α_IIb_β_3_ on the surface of platelets had been demonstrated to indirectly communicate with ADGRE5 on the surface of PCa cells during platelet-stimulated invasion^22^. However, the platelet-side signaling partner that directly binds and activates ADGRE5 had not been ascertained. Possible candidates known to bind ADGRE5 (see review^28^) include CD55 (platelet RNA-Seq TPM expression value = 82^24^), CD90 (TPM = 0^24^) and integrin α_5_β_1_ (ITGA5 TPM = 25, ITGB1 TPM = 443^24^). Our findings demonstrate an CD55-ADGRE5 connection for platelet-stimulated invasion. How integrin α_IIb_β_3_ and CD55 cooperate together to stimulate ADGRE5 and PCa cell invasion awaits future investigation. Another question that has not been adequately addressed to date is whether the various activation events following platelet-cancer cell interactions are mediated by the same or different signaling axis pairs. Our findings suggest the latter as platelet activation and PCa cell invasion were mediated by the α_IIb_β_3_-FN1 and CD55-ADGRE5 signaling axes, respectively. Neither of these axes participated in platelet-induced PCa cell apoptotic resistance. Moreover, platelet-derived cytokines CCL3L1 and IL32 participated in PCa cell invasion but not apoptotic resistance. The manner of cytokine-stimulated invasion was cooperative as opposed to additive, as the combination of both cytokines was required for stimulation of invasion (either cytokine alone was insufficient). Exogenous administration of IL32 has been demonstrated to either promote or inhibit cancer cell invasion in a cell-type specific manner^52^. Hence, our findings represent the first indication that platelets may serve as a reservoir for IL32/CCL3L1-mediated oncogenic signaling in PCa cells.

Participation of the ephrin-EPH receptor and LPA-LPAR signaling axes during platelet-induced apoptotic resistance in cancer cells has not been previously reported. Forward and/or reverse ephrin-EPH receptor signaling in cancer appears to be complex, either enhancing or suppressing malignant transformation depending on the repertoire of expressed ligands and receptors^53^. Short hairpin RNA-mediated knockdown of the EPH receptor EPHA8 in unstimulated breast cancer cells has been demonstrated to increase apoptosis in vitro^54^. Analogous to our observation that high EPHA1 expression in PCa was associated with worse patient survival (see Fig. 4C), high EPHA8 expression in breast tumors has been associated with poor patient prognosis^54^. Based on TCGA RNA-Seq and survival information available for 33 cancers, it is noteworthy that the association of high *EPHA1* expression with poor survival was restricted to PCa, whereas high *EPHA8* expression and poor survival was more broadly applicable to breast cancer, esophageal cancer, renal clear cell carcinoma and melanoma (Supplementary Fig. S6A). One of the richest sources of LPA is platelets, and activation of LPAR subtypes (LPAR1 to LPAR6) has been linked to tumor cell proliferation, migration, cytokine production, and angiogenesis^55^. In addition to our finding that PCa patients in the high *LPAR5* expression group had worse survival, other LPAR subtypes were associated with poor survival such as *LPAR1* in melanoma, *LPAR2* in adrenocortical carcinoma, *LPAR3* in pancreatic cancer, and *LPAR4* in renal papillary carcinoma (Supplementary Fig. S6B). Consequently, future studies are warranted investigating the role of both EPH receptor and LPAR signaling in the progression of different malignancies resulting from platelet-tumor cell interactions.

Platelet-stimulated MDA PCa 2b and RC77 T/E cell invasion via CD55-ADGRE5 signaling and apoptotic resistance via ephrin-EPH receptor and LPA-LPAR signaling were independent of the androgen receptor. Hence, participation of these signaling axes in platelet-stimulated oncogenesis may generalize to other cancer types as opposed to being PCa specific. Future screening of additional androgen receptor-positive PCa cell lines (e.g. LNCaP, VCaP, DUCaP, 22Rv1)^56^ that have been treated with bicalutamide, as well as assaying androgen receptor-negative cell lines from other cancer types, will help clarify this point. Also, whether platelets stimulate other hallmarks of cancer in PCa cells (e.g., escape from immune surveillance, dysregulated energetics) remains to be elucidated.

Limitations of this study should be considered. The in vitro findings of this study would benefit from orthogonal approaches such as animal models of tumor growth. For example, administration of an anti-GPIb antibody to deplete platelets in athymic nude mice partially inhibits SKOV3 human ovarian cancer xenograft growth^57^. Likewise, depleting platelets with an anti-α_2b_β_3_ antibody suppresses HT168M1 human melanoma xenograft growth in nude mice^58^. Hence, future mouse experiments should investigate the consequence of abrogating the ephrin-EPH receptor and/or LPA-LPAR signaling axes on MDA PCa 2b and RC77 T/E xenograft growth. Moreover, mouse models have demonstrated that interactions of platelets with numerous cancer cell types (e.g. mammary, colorectal, ovarian, breast) are crucial for cancer metastasis^22,59,60^. A natural corollary is to evaluate whether the presence of CD55 and/or ADGRE5 neutralizing antibodies abrogates PCa cell-platelet crosstalk and reverses metastatic potential in a nude mouse model. Another limitation to our study is the inherent drawback of a 2D culture system as well as not recapitulating the cellular diversity of the tumor. Hence, future studies should employ a 3D organoid culture system utilizing patient-derived explants to overcome these barriers. Lastly, our study has focused on the investigation of PCa-platelet interactions. It should be emphasized that the vascular microenvironmental niche encountered by PCa cells is likely complex, comprising interactions with numerous immune-related cells such as cancer-associated fibroblasts and granulocytes^61,62^.

In conclusion, our data shed light on PCa cell-platelet interactions contributing to multiple physiological events that are mechanistically facilitated by distinct receptor-ligand axis pairings. The α_IIb_β_3_-FN1 signaling axis was responsible for PCa cell-stimulated calcium mobilization in platelets. *FN1* is > 60-fold overexpressed in PCa compared to normal prostate. The α_IIb_β_3_-ADGRE5, IL32-IL32 receptor (identity of IL32 receptor remains obscure^52^) and CCL3L1-CCR1 signaling axes participated in platelet-stimulated PCa cell invasion. Noteworthy is the finding that high expression of *CCR1* in PCa was associated with significantly worse patient survival. Lastly, the ephrin-EPH receptor and LPA-LPAR signaling axes participated in platelet-induced apoptotic resistance, and high expression of *EPHA1* and *LPAR5* was associated with worse survival in PCa patients. Further investigation of these signaling axes and others could elucidate novel therapeutic strategies to mitigate both PCa progression and cancer-associated thrombosis. However, such strategies are likely to be complex given the participation of a diverse repertoire of receptors and ligands.

## Supplementary Information

Supplementary Information 1.

Supplementary Information 2.

## Data Availability

The platelet RNASeq dataset^24^ generated and analyzed during the current study are available in the ACCOuNT Data Commons Portal repository (https://acct.bionimbus.org; under the link "Discovery_Heathy_Platelet"). The PCa cell line RNASeq dataset generated during the current study are available in the Gene Expression Omnibus (GEO) repository (https://www.ncbi.nlm.nih.gov/geo/; GEO Accession number GSE216942).
