# Supplementary material for: Prostate cancer cell-platelet bidirectional signaling promotes calcium mobilization, invasion and apoptotic resistance via distinct receptor-ligand pairs
Source: Sci Rep. 2023 Feb 17;13:2864. doi: 10.1038/s41598-023-29450-x (PMC9938282; doi:10.1038/s41598-023-29450-x)
Supplement: Supplementary file 1 — Supplementary Information 1. [file 41598_2023_29450_MOESM1_ESM.pdf]

SUPPLEMENTARY FIGURES

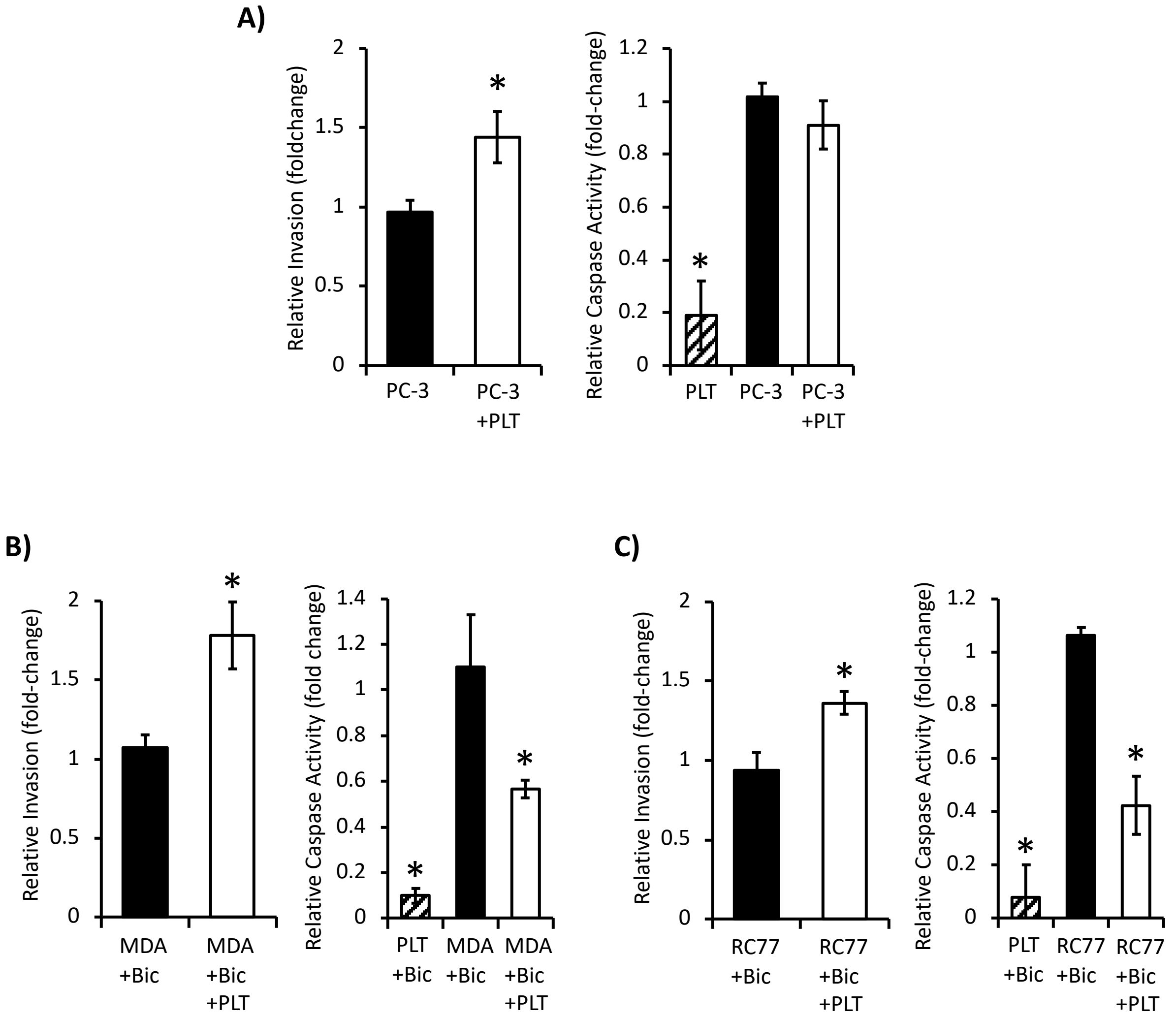

Supplementary Fig. S1. Platelet-stimulated PCa cell invasion and apoptotic resistance are independent of the androgen receptor. PC-3 cells are androgen receptor-negative. MDA PCa 2b (MDA) and RC77 T/E (RC77) are androgen receptor-positive cell lines **A)** Platelets (PLT) promote PC-3 prostate cancer cell invasion (left panel) but not apoptotic resistance (right panel). Data presented as the mean $\pm$ SEM of n = 3-4 independent determinations and analyzed by Student's t-test. *$P < 0.05$, significantly different from PC-3 (closed bar). **B)** Effect of androgen receptor blockade with 20 μM bicalutamide (Bic) on PLT-stimulated MDA cell invasion and apoptotic resistance. Data presented as the mean $\pm$ SEM of n = 4 independent determinations and analyzed by ANOVA and Dunnett's *post-hoc* test. *$P < 0.05$, significantly different from MDA + Bic (closed bar). **C)** Effect of androgen receptor blockade with 20 μM Bic on PLT-stimulated RC77 cell invasion and apoptotic resistance. Data presented as the mean $\pm$ SEM of n = 4-5 independent determinations and analyzed by ANOVA and Dunnett's *post-hoc* test. *$P < 0.05$, significantly different from RC77 + Bic (closed bar). Cell:platelet ratio for all experiments was 1:1000.

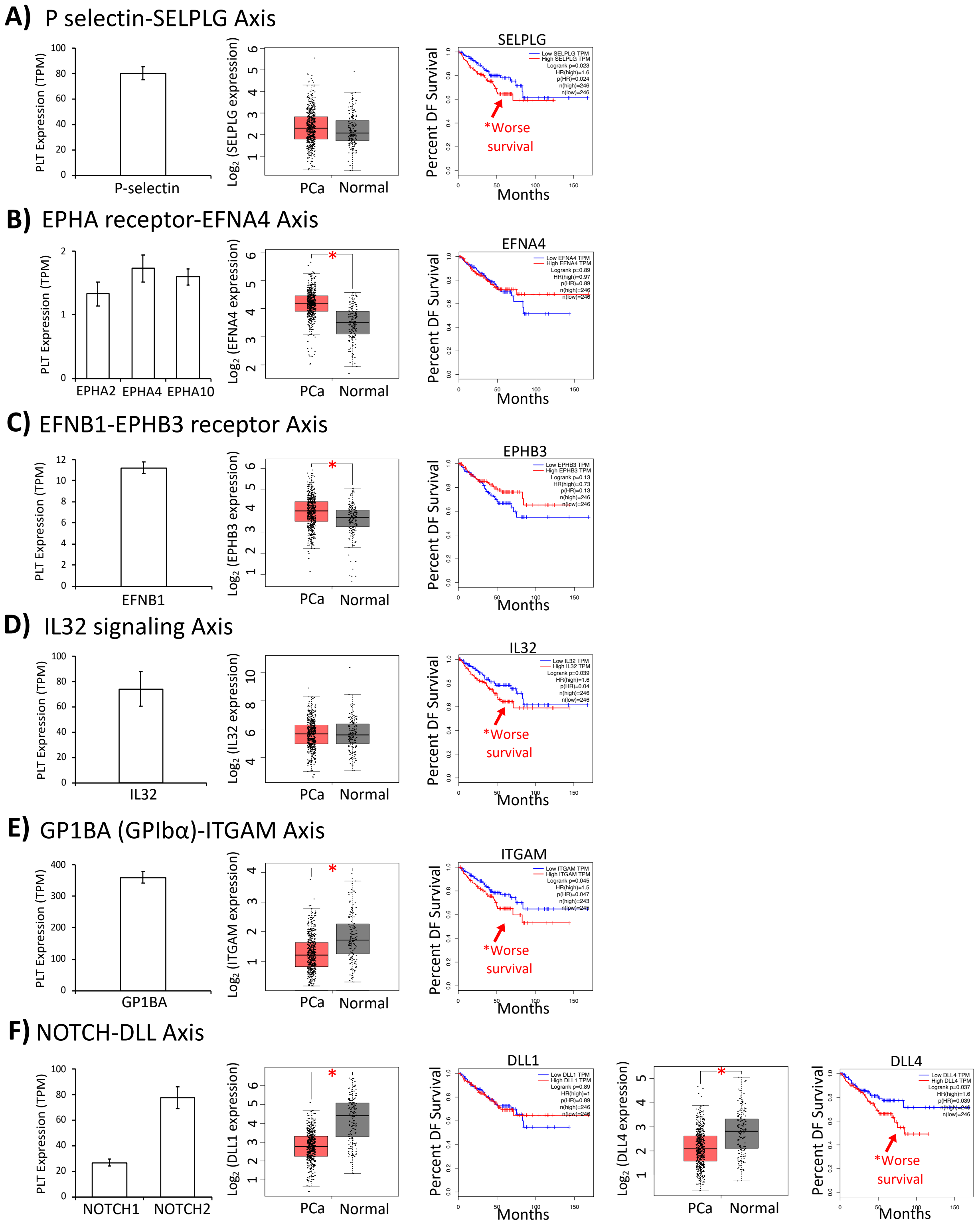

A) P selectin-SELPLG Axis
PLT Expression (TPM)
P-selectin
Log2 (SELPLG expression)
PCa
Normal
SELPLG
Percent DF Survival
*Worse survival
Months
B) EPHA receptor-EFNA4 Axis
PLT Expression (TPM)
EPHA2
EPHA4
EPHA10
Log2 (EFNA4 expression)
*
PCa
Normal
EFNA4
Percent DF Survival
Months
C) EFNB1-EPHB3 receptor Axis
PLT Expression (TPM)
EFNB1
Log2 (EPHB3 expression)
*
PCa
Normal
EPHB3
Percent DF Survival
Months
D) IL32 signaling Axis
PLT Expression (TPM)
IL32
Log2 (IL32 expression)
PCa
Normal
IL32
Percent DF Survival
*Worse survival
Months
E) GP1BA (GPIbα)-ITGAM Axis
PLT Expression (TPM)
GP1BA
Log2 (ITGAM expression)
*
PCa
Normal
ITGAM
Percent DF Survival
*Worse survival
Months
F) NOTCH-DLL Axis
PLT Expression (TPM)
NOTCH1
NOTCH2
Log2 (DLL1 expression)
*
PCa
Normal
DLL1
Percent DF Survival
Months
Log2 (DLL4 expression)
*
PCa
Normal
DLL4
Percent DF Survival
*Worse survival
Months

G) HAVCR2-LGALS9 and HAVCR2-CEACAM1 Axes

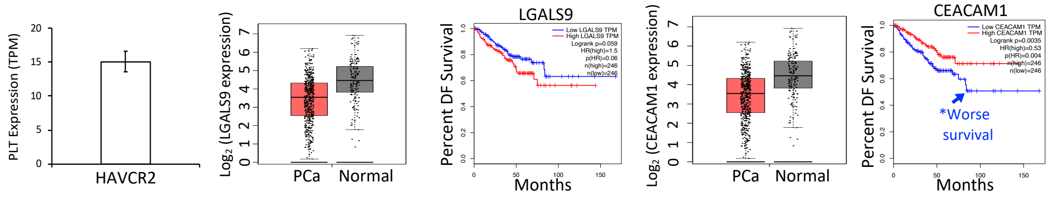

H) NRG1-ERBB3 receptor Axis

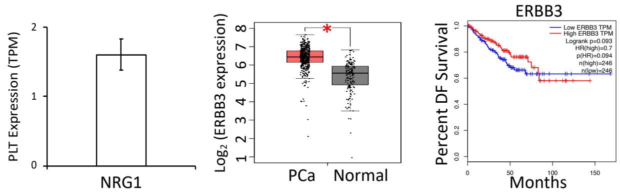

I) Integrin α6β1-laminin Axis

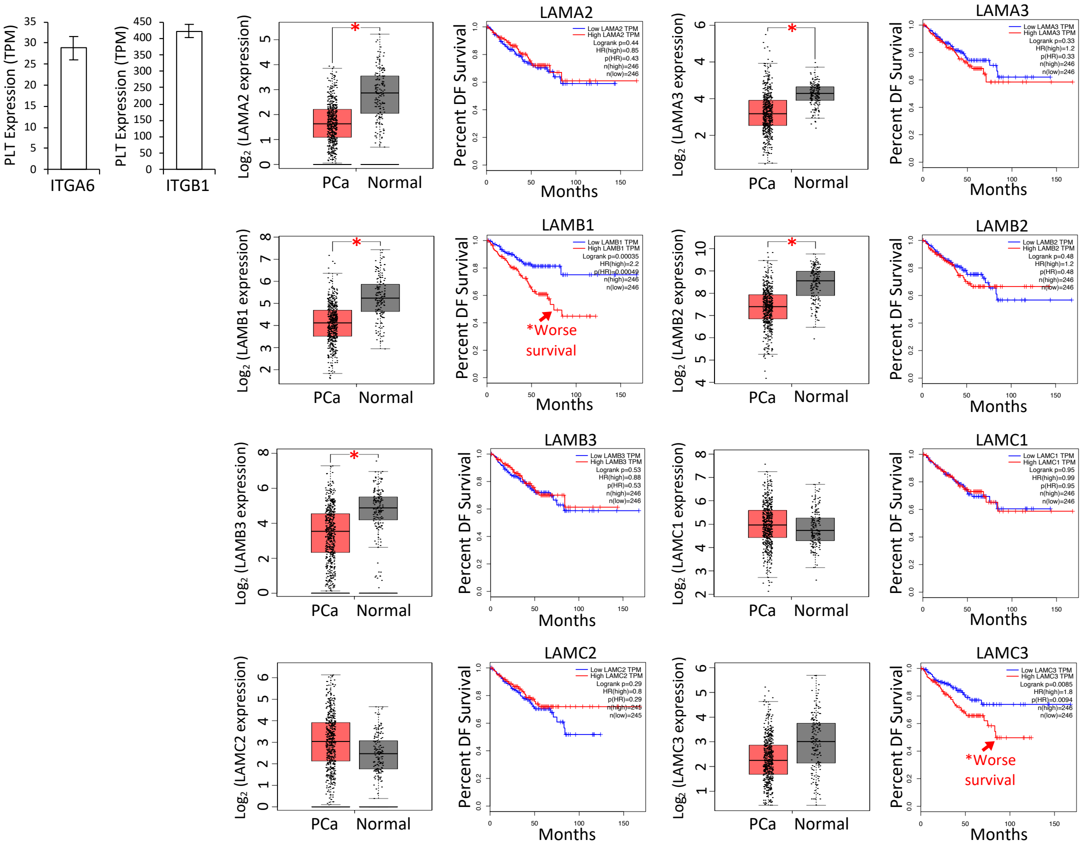

Supplementary Fig. S2. Candidate platelet-PCa signaling axis partners. Bar graphs depict expression of platelet signaling partner and corresponding PCa signaling partner based on Garofano et al. [24] and TCGA RNA-Seq data. Disease free (DF) survival plots are depicted for PCa signaling partners based on TCGA data. Signaling axes (platelet component-PCa component) displayed include **A)** P selectin-Selectin P ligand (SELPLG), **B)** EPHA receptor-EFNA4, **C)** EFNB1-EPHB3 receptor, **D)** IL32-IL32 receptor (identity of IL32 receptor remains obscure[48]), **E)** GP1BA-ITGAM, **F)** NOTCH-DLL, **G)** HAVCR2-LGALS9 and HAVCR2-CEACAM1, **H)** NRG1-ERBB3 receptor, and **I)** Integrin α6β1-laminin. TPM, transcript per million; *, Student's t-test or logrank test, *$P < 0.05$.

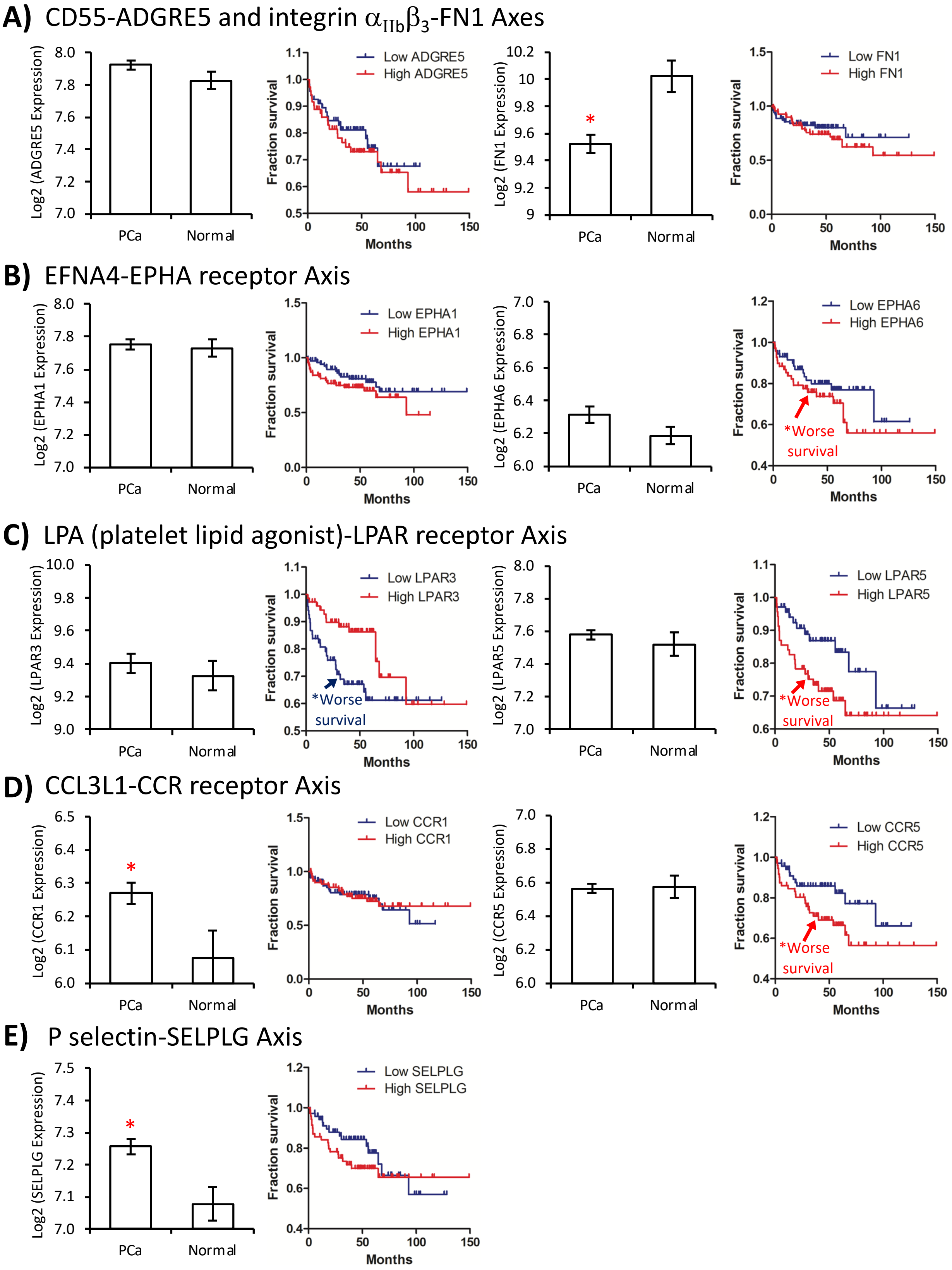
A) CD55-ADGRE5 and integrin αIIbβ3-FN1 Axes
Log2 (ADGRE5 Expression)
PCa
Normal
Low ADGRE5
High ADGRE5
Fraction survival
Months
Log2 (FN1 Expression)
*
Low FN1
High FN1
B) EFNA4-EPHA receptor Axis
Log2 (EPHA1 Expression)
Low EPHA1
High EPHA1
Log2 (EPHA6 Expression)
Low EPHA6
High EPHA6
*Worse survival
C) LPA (platelet lipid agonist)-LPAR receptor Axis
Log2 (LPAR3 Expression)
Low LPAR3
High LPAR3
*Worse survival
Log2 (LPAR5 Expression)
Low LPAR5
High LPAR5
*Worse survival
D) CCL3L1-CCR receptor Axis
Log2 (CCR1 Expression)
*
Low CCR1
High CCR1
Log2 (CCR5 Expression)
Low CCR5
High CCR5
*Worse survival
E) P selectin-SELPLG Axis
Log2 (SELPLG Expression)
*
Low SELPLG
High SELPLG

F) EPHA receptor-EFNA4 Axis

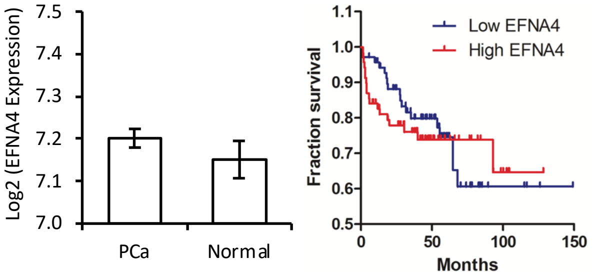

G) EFNB1-EPHB3 receptor Axis

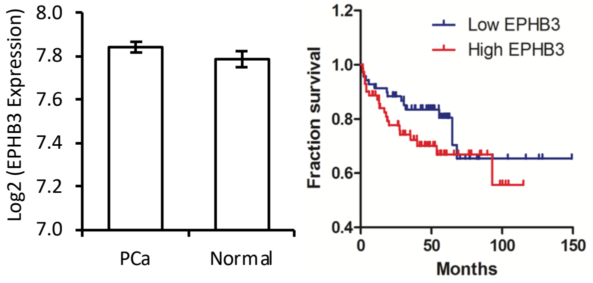

H) IL32 signaling Axis

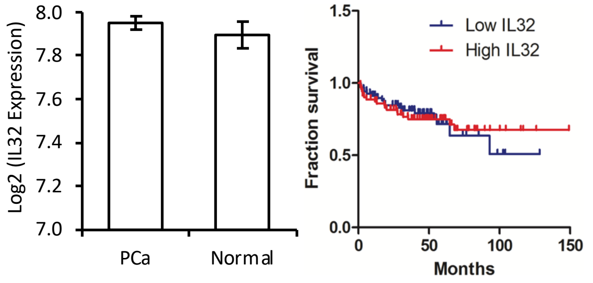

I) GP1BA (GPIbα)-ITGAM Axis

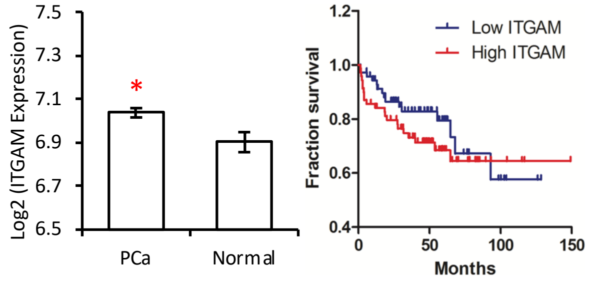

J) NOTCH-DLL Axis

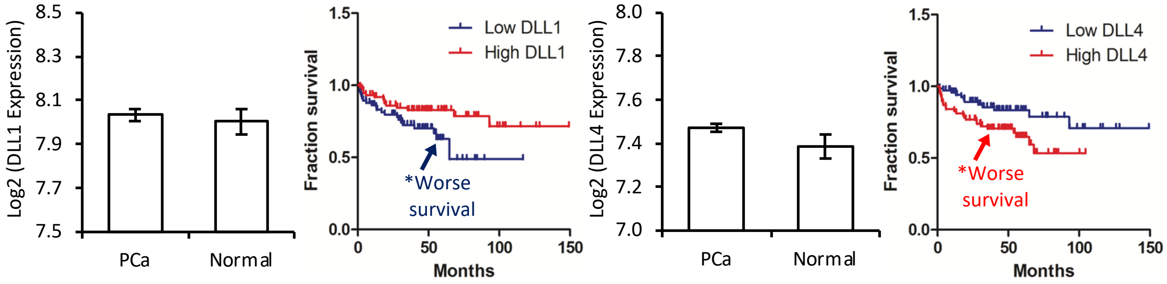

K) HAVCR2-LGALS9 and HAVCR2-CEACAM1 Axes

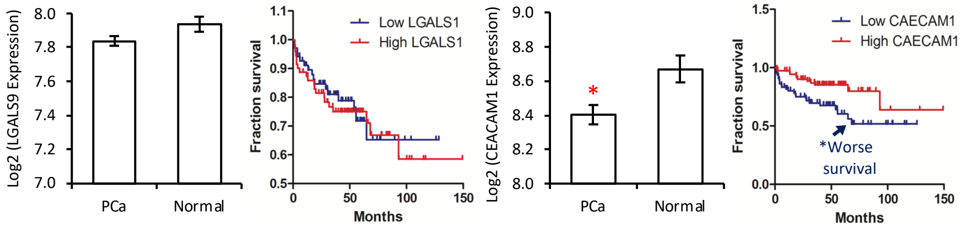

L) NRG1-ERBB3 receptor Axis

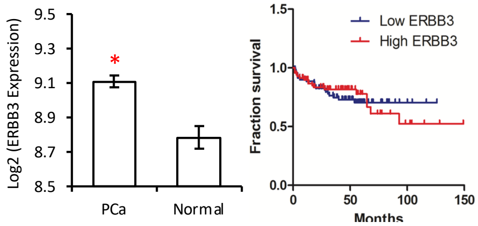

M) Integrin $\alpha6\beta1$-laminin Axis

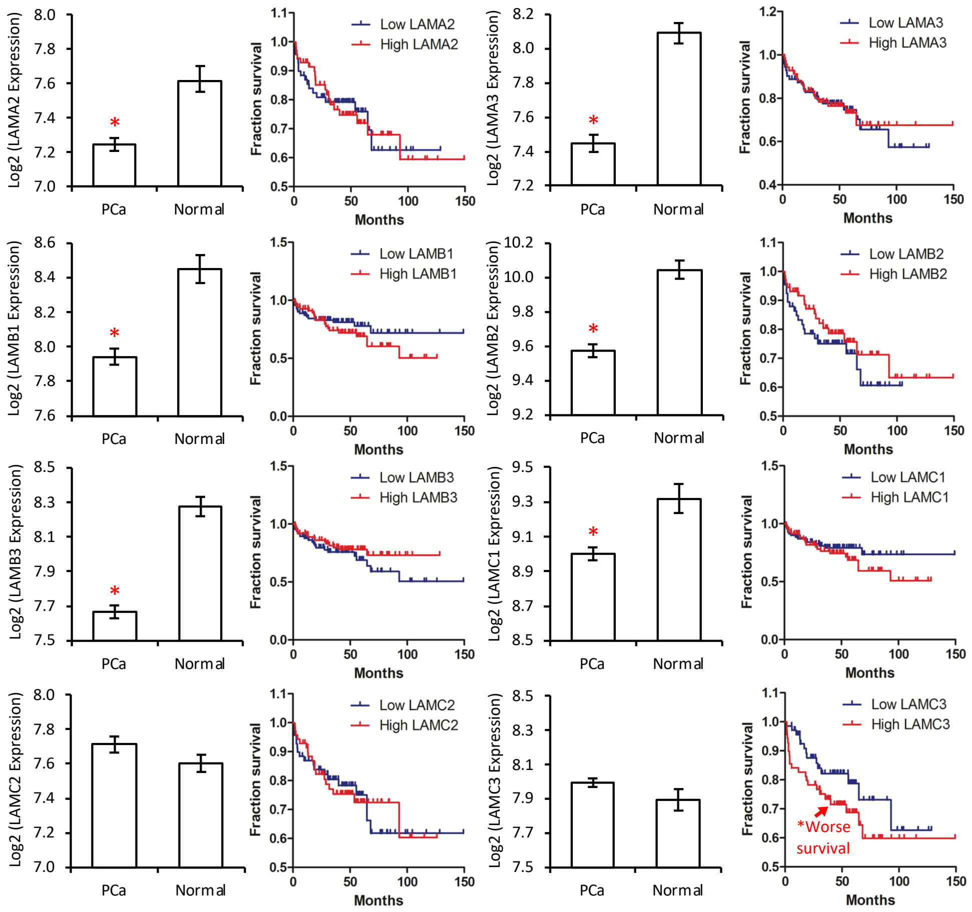

Supplementary Fig. S3. PCa signaling axis partner expression and survival analyses in separate cohort of PCa patients. Bar graphs depict expression of PCa signaling partner based on analysis of Taylor et al. Affymetrix Human Exon 1.0 ST array dataset[27]. Disease free (DF) survival plots are depicted for PCa signaling partners based on analysis of Taylor et al.[27] dataset. Signaling axis components are indicated as platelet component-PCa component. For example, CD55-ADGRE5 indicates CD55 component on platelets signaling with ADGRE5 component on PCa cells. *, Student's t-test or logrank test, *P < 0.05.

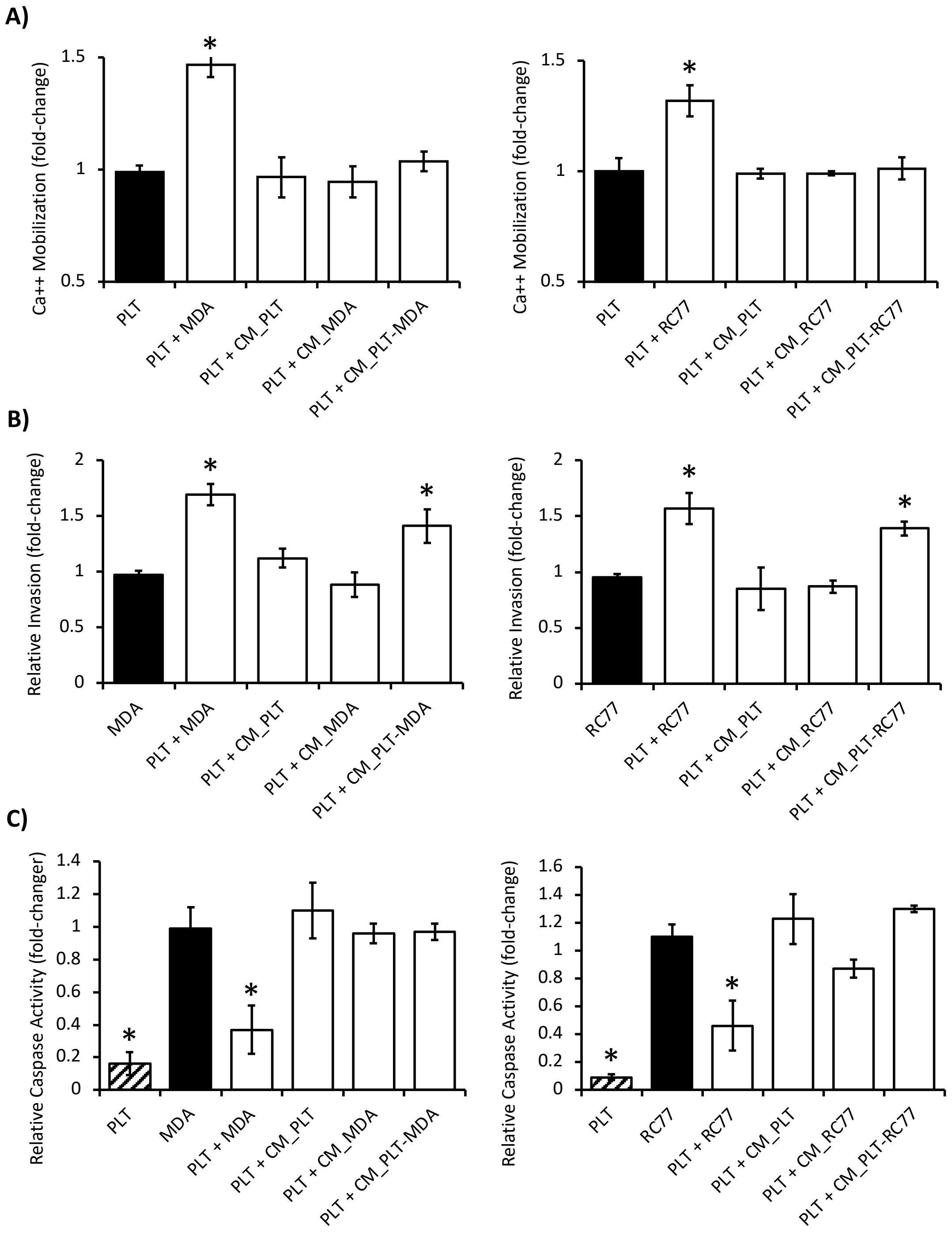

Supplementary Fig. S4. Effect of conditioned medium on platelets and PCa cells. A) Platelets (PLT) were incubated with PCa cells (MDA or RC77), or conditioned media from PLTs cultured alone (CM_PLT), PCa cells cultured alone (CM_MDA, CM_RC77) or PLTs + PCa cells cocultured (CM_PLT-MDA, CM_PLT-RC77) for 90 minutes and calcium mobilization in PLTs was measured. **B)** Same as panel A except conditioned medium was collected at 48 hours for invasion assay. **C)** Same as panel A except conditioned medium was collected at 24 hours for caspase activity assay. Data presented as the mean $\pm$ SEM of n = 3-4 independent determinations and analyzed by ANOVA and Dunnett's *post-hoc* test. *P < 0.05, significantly different from PLT group (closed bar) in panel A. *P < 0.05, significantly different from MDA or RC77 cells (closed bars) in panels B and C. Cell:platelet ratio for all experiments was 1:1000.

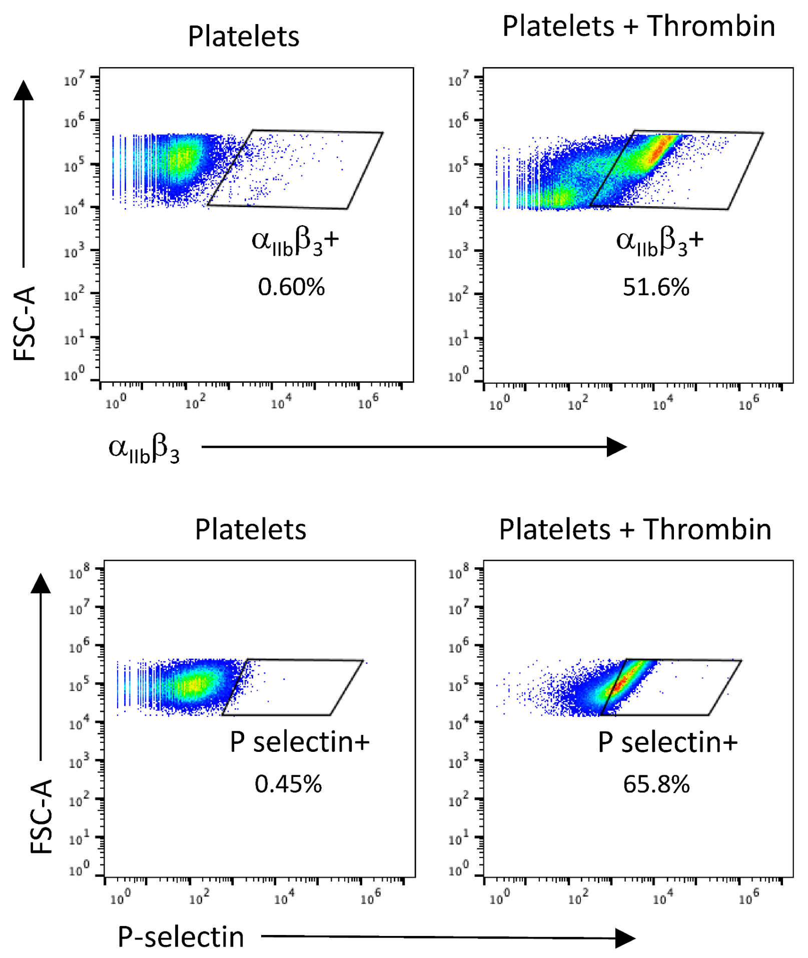

Supplementary Fig. S5. Thrombin-stimulated surface expression of P-selectin and activated $\alpha_{IIb}\beta_3$ in platelets. Platelets were activated with 0.5 units/ml thrombin for 5 minutes. Representative flow cytometry dot plots for surface expression of two platelet activation markers, P-selectin (CD62P) and activated integrin $\alpha_{IIb}\beta_3$ (PAC-1). Dot plots are representative of 3 independent assays.

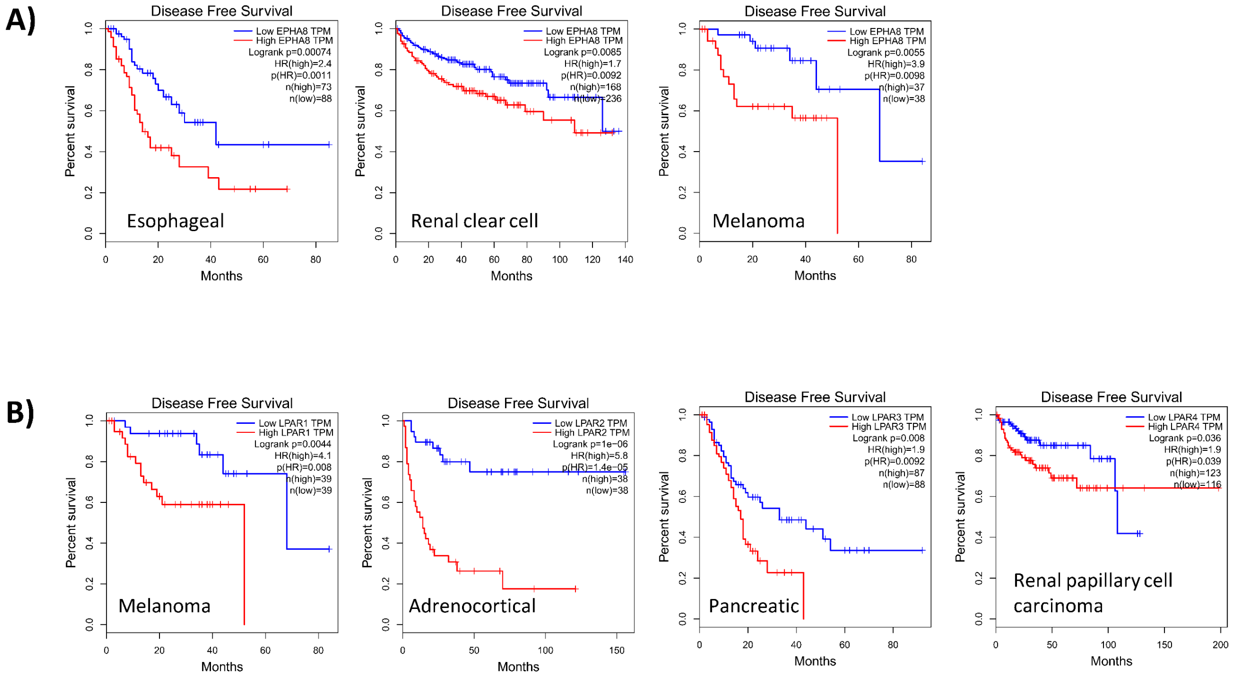

Supplementary Fig. S6. Survival plots for cancer patients with high and low expression of EPHA8 receptor or LPAR subtypes. RNA-Seq and disease-free survival data for the indicated cancers were obtained from The Cancer Genome Atlas (TCGA) (https://tcga-data.nci.nih.gov/tcga/). P-values for survival curves were determined by the logrank test. **A)** Survival plots for ephrin type-A receptor 8 (EPHA8). **B)** Survival plots for the lysophosphatidic acid receptors (LPAR1, 2, 3 and 4).
